# Phylogeny of the subtribe Asterinae (Asteraceae: Astereae): new circumscription for Asterinae and *Aster* and implications for pappus evolution

**DOI:** 10.1093/aob/mcag225

**Published:** 2026-07-25

**Authors:** Guojin Zhang, Haihua Hu, Zhixi Fu, Guohao Niu, Bohan Jiao, Caifei Zhang, Meng Wei, Goro Kokubugata, Akiko Soejima, Jian Wang, Manana Khutsishvili, Xuejun Ge, Jiahao Shen, John C Semple, Luc Brouillet, Tiangang Gao

**Affiliations:** State Key Laboratory of Plant Diversity and Specialty Crops, Institute of Botany, Chinese Academy of Sciences, Beijing 100093, China; Hunan Research Center of the Basic Discipline Developmental Biology, College of Life Sciences, Hunan Normal University, Changsha, Hunan 410081, China; State Key Laboratory of Plant Diversity and Specialty Crops, Institute of Botany, Chinese Academy of Sciences, Beijing 100093, China; State Key Laboratory of Plant Diversity and Specialty Crops, Institute of Botany, Chinese Academy of Sciences, Beijing 100093, China; College of Life Sciences, Sichuan Normal University, Chengdu, Sichuan 610066, China; State Key Laboratory of Plant Diversity and Specialty Crops, Institute of Botany, Chinese Academy of Sciences, Beijing 100093, China; College of Life Sciences, University of Chinese Academy of Sciences, Beijing 100049, China; China National Botanical Garden, Beijing 100093, China; State Key Laboratory of Plant Diversity and Specialty Crops, Institute of Botany, Chinese Academy of Sciences, Beijing 100093, China; College of Life Sciences, University of Chinese Academy of Sciences, Beijing 100049, China; China National Botanical Garden, Beijing 100093, China; State Key Laboratory of Plant Diversity and Specialty Crops, Institute of Botany, Chinese Academy of Sciences, Beijing 100093, China; State Key Laboratory of Plant Diversity and Specialty Crops, Institute of Botany, Chinese Academy of Sciences, Beijing 100093, China; Guangxi Forestry Research Institute, Nanning 530002, Guangxi, China; Department of Botany, National Museum of Nature and Science, Amakubo 4-1-1, Tsukuba, Ibaraki 305-0005, Japan; Faculty of Advanced Science and Technology, Kumamoto University, 2-39-1 Kurokami, Chuo-ku, Kumamoto 860-8555, Japan; Queensland Herbarium, Department of Environment and Science, Brisbane Botanic Gardens, Mt Coot-tha Road, Toowong, Queensland 4066, Australia; National Herbarium of Georgia, Ilia State University, Tbilisi 0105, Georgia; Center of Conservation Biology, South China Botanic Garden, Chinese Academy of Sciences, Guangzhou 510650, China; State Key Laboratory of Plant Diversity and Specialty Crops, Institute of Botany, Chinese Academy of Sciences, Beijing 100093, China; Herbarium, Institute of Botany, Jiangsu Province and Chinese Academy of Sciences, Nanjing, Jiangsu 210014, China; Department of Biology, University of Waterloo, Waterloo, Ontario N2L 3G1, Canada; University Herbier Marie-Victorin, Institut de Recherche en Biologie Végétale, Université de Montréal, 4101, rue Sherbrooke est, Montréal, Québec H1X 2B2, Canada; State Key Laboratory of Plant Diversity and Specialty Crops, Institute of Botany, Chinese Academy of Sciences, Beijing 100093, China; College of Life Sciences, University of Chinese Academy of Sciences, Beijing 100049, China; China National Botanical Garden, Beijing 100093, China

**Keywords:** *Aster*, Asterinae, classification, convergent evolution, pappus, phylogeny, RADseq

## Abstract

**Background:**

The subtribe Asterinae (Asteraceae: Astereae) comprises ∼300 species across 13 genera (including the well-known type genus *Aster*) with considerable horticultural and ecological importance. The pappus, a key character for dispersal and protection, is highly diverse and taxonomically important in Asterinae. However, the phylogenetic relationships within Asterinae and *Aster* remain poorly resolved, leading to persistent taxonomic controversies and obscuring the evolutionary history of the pappus morphology.

**Methods:**

We reconstructed both plastid (using five newly developed markers) and nuclear [using 130 503 single nucleotide polymorphisms (SNPs) generated from double digest RAD sequencing] trees, based on a comprehensive sampling of 119 species representing all 13 genera of Asterinae and allied subtribes. Using these newly obtained trees, we inferred the evolutionary trajectories of pappus morphology across Asterinae.

**Key Results:**

Both plastid and nuclear SNP trees resolved relationships within Asterinae with strong support. The previously defined Asterinae and *Aster* were revealed to be polyphyletic, splitting into four (plastid tree) or seven (nuclear SNP tree) clades, respectively. Both trees strongly supported a large clade containing most genera and species of the previously circumscribed Asterinae. Within this large clade, four consistent and highly supported subclades were recovered, while three additional clades were resolved outside of it. Incongruent placements of several taxa between the two trees were detected. Ancestral state reconstruction revealed extensive homoplasy in pappus evolution, undermining the taxonomic reliability of pappus morphology.

**Conclusions:**

We redefine Asterinae to include only its core clade and recognize a single monophyletic *Aster*. This revised taxonomy of Asterinae consolidates 16 former genera of Astereae. Additionally, we propose one new subtribe (Chlamyditinae) and four subgenera within *Aster* – three of which are novel. This framework clarifies the taxonomy of Asterinae and *Aster* and lays the groundwork for future studies on the evolution of the pappus and other characters that may drive the rise of its remarkable species diversity.

## INTRODUCTION

The evolution of fruits in flowering plants represents a key innovation that enhances seed protection while enabling adaptive dispersal by specialized morphological structures. Distinct fruit characters have evolved in response to specific dispersal vectors: fleshy fruits develop nutritive pulp to attract animals, the samaras form aerodynamic wings for wind transport and hydrochorous fruits acquire specialized aerenchymatous tissues for aquatic flotation (Van der Pijl, 1982; Seale and Nakayama, 2020; Xiang *et al*., 2024). These fruit structures increase the dispersal distance, reduce the competition among individuals and help the seeds reach more niches – collectively serving as evolutionary drivers that may have contributed significantly to angiosperm diversification (Van der Pijl, 1982; Seale and Nakayama, 2020; Xiang *et al*., 2024; Zhang and Ma, 2024). Asteraceae is one of the largest angiosperm families with ∼30 000 species, characterized by a capitulum – an inflorescence composed of one or more types of small flowers (florets) (Anderberg *et al*., 2007; Funk *et al*., 2009; Zhang *et al*., 2024). Most Asteraceae species have evolved a variously modified persistent calyx, namely pappus, to help in the dispersal of achenes, such as capillary, scaly or awn-like pappus, which might contribute to the remarkable species diversity of the family (Jeffrey, 2009). The achenes of most Asteraceae are crowned by a capillary pappus of bristles, becoming parachute fruits that can be dispersed far by wind (Sheldon and Burrows, 1973; Van der Pijl, 1982). In addition, in some Asteraceae species, the pappus protects achenes from UV radiation and cold stress, and also facilitates their water uptake (Karabourniotis *et al*., 1995, 2020; Shaukat *et al*., 2004; Yan *et al*., 2012). Furthermore, in many types of pappus, the shape and length of bristles are variable across species, and these pappus characters are often considered to be conserved and employed in the taxonomy of many groups in Asteraceae (Anderberg *et al*., 2007; Funk *et al*., 2009).

The tribe Astereae is one of the largest tribes of Asteraceae, containing over 3000 species in ∼250 genera (Nesom and Robinson, 2007; Nesom, 2020*a*). Previously, 18–36 subtribes have been proposed within Astereae and the circumscriptions of many subtribes have long been controversial (Nesom and Robinson, 2007; Brouillet *et al*., 2009; Nesom, 2020*a*). Asterinae, a large subtribe of Astereae, contains 280–500 species in >10 genera depending on taxonomic treatment (Zhang and Bremer, 1993; Nesom, 1994*a*; Nesom and Robinson, 2007). It comprises many species of economic and cultural value: ornamental plants [e.g. asters and *Callistephus chinensis* (L.) Nees], vegetables [e.g. *Kalimeris indica* (L.) Sch.Bip. and ‘*Doellingeria*’ *scabra* (Thunb.) Nees (the double quotation marks indicate generic placements or combination names that are not accepted in the present study)] and traditional medicinal plants (e.g. *Aster tataricus* L.f. and *A. turbinatus* S.Moore). Morphological characters, such as habit, leaves, floret colour, phyllaries and pappus, are highly variable in Asterinae, resulting in the controversial circumscription of the subtribe itself and its constituent genera.

The current circumscription of Asterinae can be traced back to the earlier subtribal classification of Astereae, which was based primarily on morphological evidence (Nesom, 1994*a*). This initial circumscription included 14 genera and 306 species – most of which are distributed in the Old World. *Boltonia* L’Hér, a genus native to the New World, was first included in Asterinae (Nesom, 1994*b*), but later excluded in a subsequent revision of Astereae (Nesom and Robinson, 2007). This exclusion restricted Asterinae to 13 genera and ∼280 species. More recently, a modified circumscription of Asterinae was proposed (Nesom, 2020*a*), which excluded additional genera (e.g. *Galatella* Cass.) and included others (e.g. *Myriactis* Less.). These successive revisions indicated that the circumscription of Asterinae remains controversial.

Similarly, the delimitation of genera within Asterinae has long been disputed, particularly for the well-known and species-rich genus *Aster*. Based primarily on achene morphology and cytology, Nesom (1994*b*) restricted *Aster* to the Old World, adopting a narrow circumscription of *Aster* (*Aster s.s.*) that contained ∼180 species and excluded many segregated genera [e.g. *Kemulariella* Tamamsch., *Conyzanthus* Tamamsch., *Doellingeria* Nees, *Kalimeris* Cass., *Asterothamnus* Novopokr., *Rhinactinidia* Novopokr. (‘*Krylovia*’), *Turczaninovia* DC., *Galatella* Cass., *Crinitina* Soják (‘*Linosyris*’) and *Tripolium* L.]. This strict circumscription of *Aster* was accepted by many studies (Tamamschjan, 1959; Ling, 1985; Li *et al*., 2012; Zhang *et al*., 2015, 2019). By contrast, other taxonomists favoured a broad circumscription of *Aster* (*Aster s.l.*) that included the majority of all segregated genera and contained ∼300 species (Grierson, 1975; Merxmüller *et al*., 1976; Ito and Soejima, 1995; Chen *et al*., 2011). For instance, in *Flora Europaea* (Merxmüller *et al*., 1976), members of the *Galatella* group (including *Galatella*, *Tripolium* and *Crinitina*) were included in *Aster*. Recently, several new genera have been segregated from *Aster* based primarily on morphological characters. For example, the African *Aster*, containing *Aster bakerianus* Burtt Davy ex C.A.Sm. and its 17 relatives distributed in sub-Saharan Africa, was treated as *Afroaster* J.C.Manning & Goldblatt (Manning and Goldblatt, 2012), a genus phylogenetically distant from Asterinae, and more than ten new genera were proposed for the Asian *Aster* species (Nesom, 2020*a*). However, phylogenetic relationships between these segregated genera and the core *Aster* have not been comprehensively investigated, leaving genus-level delimitations within Asterinae unresolved.

Early phylogenetic studies using ITS sequences (Noyes and Rieseberg, 1999; Fiz *et al*., 2002) revealed that New World groups and the *Bellis* group were distantly related to the Old World Asterinae (including the type genus *Aster*), supporting the separation of subtribes Symphyotrichinae and Bellidinae from Asterinae. Another ITS-based study clarified the phylogenetic placements of *Olearia* and suggested the existence of an Australasian lineage that might be related to Asterinae (Cross *et al*., 2002). A synthesis of previous ITS datasets for Astereae further indicated that Asterinae is non-monophyletic and nested within the Australasian clade, providing new insight into the evolutionary history of the subtribe (Brouillet *et al*., 2009). Subsequent studies (Li *et al*., 2012; Jafari *et al*., 2015; Korolyuk *et al*., 2015; Farhani *et al*., 2018) used one or two plastid (*trnL-F* and *psbA-trnH*) and/or nrDNA markers (ITS and ETS), but sampled fewer than 50 *Aster* species and only a subset of segregated genera. These studies also confirmed the non-monophyly of Asterinae, which was found to nest within multiple clades of Astereae.

Phylogenetic studies have consistently resolved both *Aster s.l.* and *Aster s.s.* as non-monophyletic, thereby rejecting the morphologically circumscribed boundaries between *Aster* and its allied genera. However, previous studies failed to resolve the phylogenetic backbone of both Astereae and Asterinae due to inadequate taxon sampling and low resolution of the molecular markers used. Therefore, a comprehensive taxon sampling coupled with higher-resolution molecular markers is required – this will not only clarify the circumscription of Asterinae, but also resolve the phylogenetic relationships between *Aster* and its allies.

Single nucleotide polymorphisms (SNPs) are widely used in phylogenetic analyses of closely related genera and species (Leaché and Oaks, 2017; Jiao *et al*., 2023). Restriction site-associated DNA sequencing (RADseq) is a cost-effective and efficient approach for generating a large number of nuclear-derived SNPs (Baird *et al*., 2008; Yang *et al*., 2016). Such SNP datasets were highly effective for reconstructing nuclear phylogenies across diverse taxa, including *Carex*, bamboos and oaks, consistently yielding well-resolved phylogenies with strong statistical support (Wang *et al*., 2013; Hipp *et al*., 2014; Massatti *et al*., 2016; Fitz-Gibbon *et al*., 2017).

The pappus of Asterinae displays striking morphological diversity, including long pappus (as long as the disc floret corolla), short pappus (shorter than that of the disc floret corolla), dimorphic pappus (short in ray floret and long in disc floret), double pappus (outer row of scales and inner row of bristles) (Fig. 1) and pappus lacking (epappose). This diversity has established pappus type a key taxonomic character in Asterinae. For example, *Kalimeris*, *Miyamayomena* Kitam. and *Heteropappus* Less. were distinguished from other genera by short pappus, epappose achenes and a dimorphic pappus, respectively. Yet, species sharing the same pappus type often differ significantly in other morphological characters, leading to their placement in distinct genera. For example, ‘*Doellingeria*’ *marchandii* (Levl.) Ling. (=*Aster marchandii* Lévl.), ‘*D*’*. japonica* (Miq.) G.L.Nesom [=*Aster miquelianus* H.Hara] and *Aster giraldii* Diels all have short pappus, but they were not placed in *Kalimeris* (Ling, 1985; Nesom and Robinson, 2007). Furthermore, previous phylogenetic studies (Li *et al*., 2012; Jafari *et al*., 2015; Korolyuk *et al*., 2015; Farhani *et al*., 2018) recovered species with similar pappus type in distant clades. Moreover, natural hybrids between species with different pappus types were frequently documented, such as hybrids between *Aster* and *Kalimeris* (Tara, 1972, 1978; Ito *et al*., 1995; Li 2006). These studies suggest that pappus morphology may have evolved convergently, limiting its reliability as a taxonomic character. However, the evolutionary pattern of pappus morphology in Asterinae remains poorly understood due to the lack of a well-resolved phylogenetic framework. Consequently, the utility of pappus for classifying Asterinae remains untested. A robust phylogeny with comprehensive sampling is therefore essential: it will not only advance our understanding of Asterinae systematics, but also clarify the evolutionary dynamics of pappus in this subtribe.

**Fig. 1.**
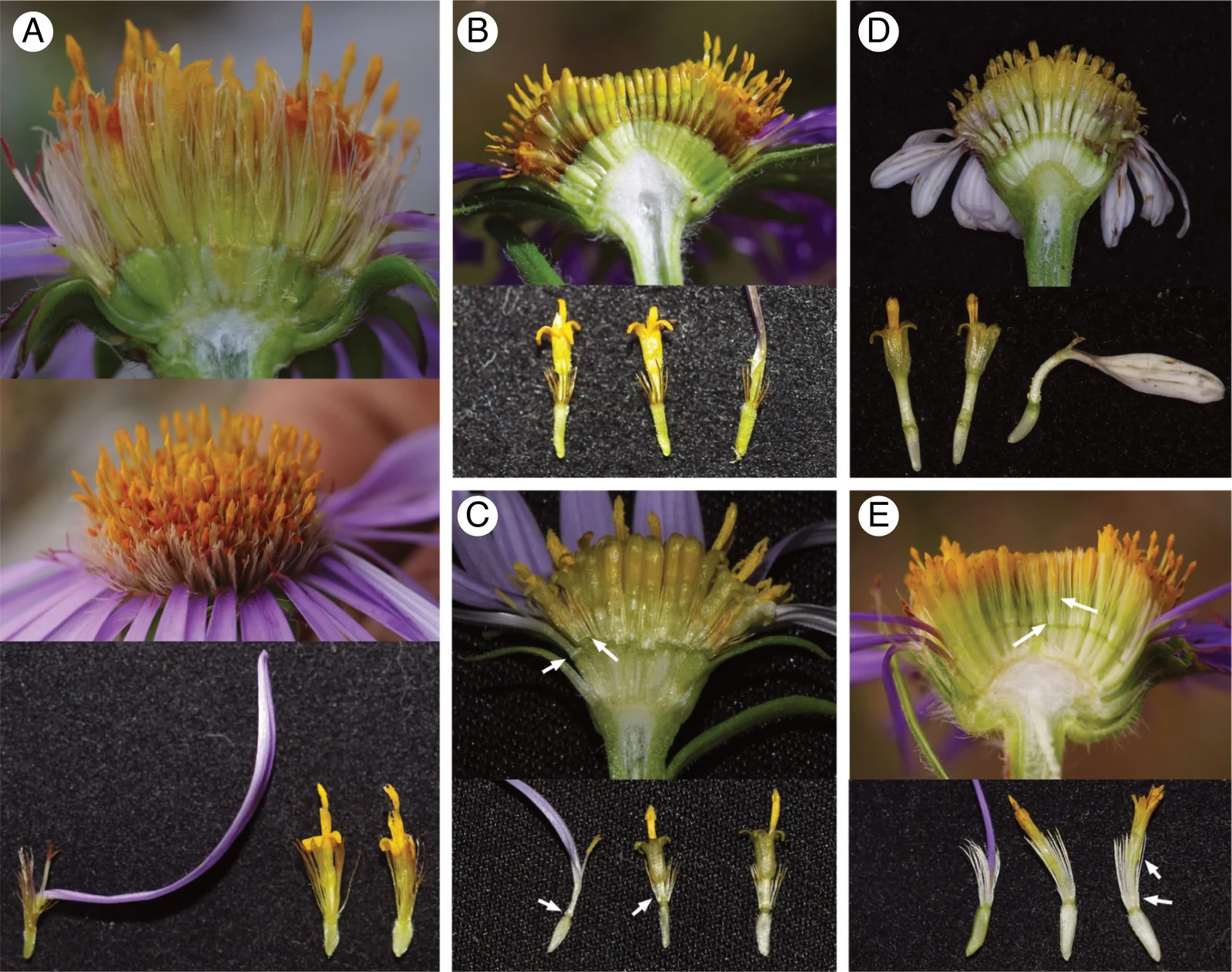
Pappus types of Asterinae. (A) Long pappus (*Aster handelii*). (B) Short pappus (*A. souliei*). (C) Dimorphic pappus (*Heteropappus arenarius*). (D) Pappus absent (*Miyamayomena angustifolia*). (E) Double pappus (*A. yunnanensis*). Arrows in panels (C) and (E) indicate distinctive pappus structures.

Here, we generated a new phylogenetic framework for Asterinae and its constituent genus *Aster* using two datasets: five highly variable plastid DNA markers (developed specifically for this study) and nuclear SNP data developed via double digest RADseq (ddRADseq). By comparing these newly obtained plastid and nuclear phylogenies, we addressed three key objectives: (1) testing the monophyly of Asterinae (subtribal monophyly) and its constituent genera (generic monophyly); (2) reconstructing the evolutionary trajectory of pappus morphology; and (3) evaluating the taxonomic utility of pappus. Integrating phylogenetic and morphological evidence, we propose a revised classification for Asterinae, including redefined generic boundaries for both Asterinae and *Aster*, and a new taxonomic synthesis that resolves decades of systematic ambiguity in this complex group.

## MATERIALS AND METHODS

### Taxonomic sampling

The previous subtribal circumscription (Nesom, 1994*a*; Nesom and Robinson, 2007) and generic delimitations within Asterinae (Nesom, 1994*b*) were followed in this study. We included 203 samples of Astereae in the plastid phylogenetic analysis and character reconstruction and used two species of Anthemideae as outgroups. All 13 genera of Asterinae with 137 samples from 114 species, including 103 samples representing 84 species of *Aster*, were sampled. Nearly all recently described genera of Asterinae (Nesom, 2020*a*) except *Iteroloba* G.L.Nesom (a monotypic genus only from the type collection) were also sampled. One species of *Neobrachyactis*, an undescribed genus (‘para-*Brachyactis*’) of Asterinae in treatments of Nesom (1994*b*) but not recognized in Nesom and Robinson (2007), was also included in the analysis. Fifty-seven non-Asterinae species belonging to unplaced groups or other subtribes of Astereae were included to investigate the monophyly of Asterinae. We sampled five species of putative ‘*Doellingeria*’ that were usually considered as close relatives of *Aster* but were treated as an unplaced group in Nesom and Robinson (2007). The monotypic genus *Turczaninowia*, considered to be a segregated genus of *Aster* by many Chinese taxonomists but treated as a member of *Aster* by Nesom (1994*b*), was also sampled. Our preliminary molecular and morphological analyses suggested that the karst shrubby genus *Heteroplexis* was closely related to *Aster*, but its phylogenetic placement was not investigated previously. We included seven samples from five species of *Heteroplexis* to investigate its molecular phylogenetic position for the first time. Detailed sampling information is listed in Supplementary Data Table S1.

For the nuclear phylogenetic analyses, we included 120 species, comprising 88 species from Asterinae covering all 13 genera recognized in Asterinae, with 69 species of these belonging to *Aster* (Supplementary Data Table S2). Additionally, we sampled 28 species from other subtribes of Astereae to test the monophyly of Asterinae, along with four species from Gnaphalieae and Anthemideae as outgroups. Similar to the plastid analyses, the nuclear phylogenetic analyses sampled five species of *Doellingeria*, one species of *Turczaninowia* and two species of *Heteroplexis*.

**Table 1.** Comparison of the delimitation and classification of Asterinae in previous studies and this study.

This study	Nesom (1994*a*) integrated information from Nesom (1994*b*), Nesom and Robinson (2007) and Ling (1985)	*Flora of China* (Chen *et al*., 2011)	Nesom (2020*a*)
***Aster* subg. *Aster***	***Aster* sect. *Aster* [excl. *A. auriculatus*, *A. falcifolius*, *A. fuscescens*, *A. poliothamnus*, *A. sikuensis* and *A. sibiricus* (=*Eurybia sibirica)*]**	*A.* sect. *Aster* [excl. *A. auriculatus*, *A. dolichophyllus*, *A. falcifolius*, *A. fuscescens*, *A. poliothamnus*, *A. sikuensis* and *A. piccolii* and relatives (species numbered 58–64 in *Flora of China*)]	***Aster***
	***Aster* sect. *Orthomeris* (excl. *A. dolichophyllus*, *A. pycnophyllus*, Ser. *Albescentes* and Ser. *Hersileoides*)**	*A*. sect. *Ageratoides*	
	***Aster* sect. *Alpigenia* (only incl. Ser. *Alpini* and Ser. *Tongolenses*)**	*A.* sect. *Alpigeni* (*A. alpinus* and relatives: species numbered 80–94 and 123 in *Flora of China*)	
	***Aster falcifolius***	*Aster falcifolius*	***Chaochienchangia***
	***Heteropappus***	*A.* sect. *Pseudocalimeris*	***Heteropappus***
	***Kalimeris* (excl. *K. longipetiolata*)**	*A.* sect. *Asteromoea* (excl. *A. longipetiolatus*)	***Kalimeris***
	***Krylovia* (*Rhinactinidia*)**	*Rhinactinidia*	***Rhinactinidia***
	***Gymnaster* (*Miyamayomena*)**	*Aster koraiensis*, *A. piccolii*, *A. pseudosimplex*, *A. savatieri* and *A. sinoangustifolius*	***Miyamayomena***
	*Heteroplexis* (unplaced)	*Heteroplexis*	*Heteroplexis* (Grangeinae)
	*Sheareria* (Lagenophorinae)	*Sheareria*	***Sheareria***
	*Rhynchospermum* (Lagenophorinae)	*Aster verticillatus*	***Rhynchospermum***
	***Turczaninovia***	*Turczaninovia*	***Turczaninovia***
***Aster* subg. *Dongyaster***	Asian *Doellingeria* (unplaced)	*A.* sect. *Teretiachaenium*	***Cardiagyris***
	***Aster dolichophyllus***	*Aster dolichophyllus*	
***Aster* subg. *Liangwan***	***Aster* Ser. *Hersileoides***	*Aster incertae sedis* group	***Yonglingia***
***Aster* subg. *Muwan***	***Aster poliothamnus* and *A. sikuensis***	*Aster poliothamnus* and *A. sikuensis*	***Sinobouffordia***
	Not included	*Aster langaoensis*	Not included
	***Asterothamnus***	*Asterothamnus*	***Asterothamnus***
	***Arctogeron***	*Arctogeron*	***Arctogeron***
*Galatella*	***Galatella***	*Galatella*	*Galatella* (Bellidinae)
	***Linosyris* (*Crinitaria*)**	*Crinitina*	*Crinitina* (Bellidinae)
*Tripolium*	***Tripolium***	*Tripolium*	*Tripolium* (Bellidinae)
*Kitamuria*	***Aster glehnii***	Not included	*Kitamuria* (Bellidinae)
Astereae unplaced groups	***Callistephus***	*Callistephus*	***Callistephus***
	***Aster* sect *Orthomeris* (only Ser. *Albescentes*)**	*Aster incertae sedis* group	***Sinosidus***
	*Kalimeris longipetiolata*	*Aster longipetiolatus*	***Cordiofontis***
	*Formania*	*Formania*	*Formania* (Formaninae)
	***Aster fuscescens***	*Aster fuscescens*	***Griersonia***
	***Aster auriculatus***	*Aster auriculatus*	***Metamyriactis*** (excluding *Aster paduratus*)
	***Aster veitchianus***	*Aster veitchianus*	
	***Aster pycnophyllus***	*Aster pycnophyllus*	
	***Psychrogeton***	*Psychrogeton*	***Psychrogeton***
	*Brachyactis* [excl. *B. ciliata* (=*Symphiotrichum ciliatum*)]	*Neobrachyactis*	***Neobrachyactis***
	***Aster bipinnatisectus***	*Aster bipinnatisectus*	***Iteroloba***
	***Kemulariella* (excl. *K. caucasica*, the type species)**	Not included	***Kemulariella***
*Kemulariella* (*Kemulariella* group)	***Kemulariella caucasica***	Not included	***Kemulariella caucasica***
*Chlamydites* (Chlamyditinae)	***Aster* sect. *Alpigenia* (excl. *A. bipinnatisectus*, *Aster batangensis*, Ser. *Alpini* and Ser. *Tongolenses*)**	*A*. sect. *Alpigeni* (species numbered 95–119 and 121 in *Flora of China*)	***Chlamydites***
	***Aster bipinnatisectus***	*Aster bipinnatisectus*	***Tibetiodes***
	***Aster batangensis***	*Aster batangensis*	***Geothamnus***
*Afroaster* (Afroasterinae)	***Aster bakerianus* and relatives**	Not included	*Afroaster* (Afroasterinae)

Bold type indicates the taxon belonging to Asterinae in the corresponding classification. No subtribe information was provided in *Flora of China*.

### DNA extraction, amplification and sequencing of plastid sequences

Total genomic DNA was extracted from 20–40 mg silica gel-dried leaf materials using a TIANGEN plant genomic DNA extraction kit (TIAN-GEN Biotech., Beijing, China) following the manufacturer's protocol. Chloroplast genomes of *Aster spathulifolius* (Choi and Park, 2015) and *Chrysanthemum indicum* (Xia *et al*., 2016) were downloaded from NCBI GenBank and the chloroplast genome of *Erigeron* (*Conyza*) *canadensis* was assembled from the whole genome data (Peng *et al*., 2014). The three chloroplast genomes were aligned using MAFFT v.7 online (Katoh *et al*., 2019) and five highly variable sequences (partial *ycf1* gene, *atpI–H*, *rpl32–trnL^(UAG)^*, *ndhF–rpl32* and *ycf3–trnS^(GGA)^*) were selected as the molecular markers to reconstruct the phylogeny of Asterinae. We designed novel primers for PCR amplification of these regions, with detailed information listed in Supplementary Data Table S3. PCRs were performed in 25-μL volumes with 21 μL of Golden Star T6 Super PCR Mix (Tsingke Biotech., Beijing, China), 2 μL of 5 pmol μL^–1^ forward primer, 2 μL of 5 pmol μL^–1^ reverse primer and 1 μL DNA template. The PCR cycles for all sequences except *ycf1* consisted of an initial incubation of 98 °C for 2 min, followed by 30 cycles of 98 °C for 10 s, 54 °C for 30 s and 72 °C for 30 s, and terminated with an incubation of 72 °C for 5 min. For the *ycf1* gene, the enolization time was extended to 1 min and other parameters were the same as described above. The purified products were sequenced by Tsingke Biotech. and Majorbio® (Shanghai, China) with the ABI 3500 sequencer and the same primers used for PCR amplification.

### Phylogenetic reconstruction using plastid sequences

Sequences were assembled using Sequencher v.4.1.4 and aligned using MAFFT v.7 online (Katoh *et al*., 2019) with manual adjustments in Bioedit 7.2.2. (Hall, 1999). The combined matrix contained 7081 aligned characters and five partitions, i.e. *ycf1-1* (1752 characters), *atpI–H* (1290 characters), *rpl32–trnL^(UAG)^* (1518 characters), *ndhF–rpl32* (1663 characters) and *ycf3–trnS^(GGA)^* (858 characters). A phylogeny was reconstructed using the maximum-likelihood (ML) method with IQ-TREE 1.5.5 (Minh *et al*., 2013; Nguyen *et al*., 2015; Chernomor *et al*., 2016; Hoang *et al*., 2018) and RAxML v.8.2.10 (Stamatakis *et al*., 2008; Stamatakis, 2014), and the Bayesian inference (BI) method with MrBayes 3.2.6 (Huelsenbeck and Ronquist, 2001; Ronquist *et al*., 2012). For both ML and BI methods, substitution models were selected based on the corrected Akaike's Information Criterion (AICc) calculated using ModelFinder in IQ-TREE (Kalyaanamoorthy *et al*., 2017). For partitioned analyses (Minh *et al*., 2013; Nguyen *et al*., 2015; Chernomor *et al*., 2016), TVM + R3 for *ycf1-1*, GTR + G4 for *atpI–H*, TVM + R3 for *rpl32–trnL^(UAG)^*, TVM + R3 for *ndhF–rpl32* and K3Pu + R3 for *ycf3–trnS^(GGA)^* were used in IQ-TREE; the GTRCAT model was used for all five markers in RAxML; and GTR + G4 was used for all segments in MrBayes. For non-partitioned analyses, GTR + R4 was used for ML analysis in IQ-TREE, GTRCAT was used for ML analysis in RAxML and GTR + G4 was used for BI analysis in MrBares. Ultrafast bootstrapping and rapid bootstrapping with 1000 replications were adopted in IQ-TREE (Minh *et al*., 2013; Nguyen *et al*., 2015) and RAxML, respectively. Bayesian inferences were conducted using MrBayes on the CIPRES Science Gateway v.3.3 (Miller *et al*., 2010) with two runs. Each run was with four chains running 20 million generations and trees were sampled every 4000 generations. Convergence of chains were checked using Tracer 1.7 (Rambaut *et al*., 2018) to ensure estimated sample size (ESS) values were greater than 200. The Bayesian posterior probabilities of each node were obtained in the two runs.

### ddRAD seq, SNP generation and nuclear phylogenetic tree reconstruction

The ddRADseq libraries were built and the subsequent sequencing was conducted in Biomark (Beijing, China) with an Illumina HiSeq X Ten sequencer following previously described procedures (Sun *et al*., 2013). The combination of RsaI + HaeIII restriction enzymes was used to digest the DNA after preliminary assessments of different combinations of enzymes. The digested DNA segments with length of 264–314 bp were retained and used in sequencing. Quality control of sequencing datasets was performed in FastQC and low-quality reads were removed (Andrews, 2010). The retained reads were used for SNP calling. First, we used BLAT to cluster all reads, and the reads with 90 % identity were assigned into one group following the procedures in the previous analysis (Kent, 2002; Sun *et al*., 2013). The read with the highest quality in each group was selected as the reference. The reads of each species were thyen mapped to the reference reads using BWA (Li and Durbin, 2009). SNPs were obtained using both GATK and SAMtools, the intersection of SNPs shared by four or more samples was retained (McKenna *et al*., 2010; Danecek *et al*., 2021), and more than one SNP was allowed for each locus. Additionally, on the basis of the number of species that shared an SNP, we generated 14 subsets of SNPs that were shared by 4–120 species (Supplementary Data Table S4). Moreover, we also used ipyrad 0.7.28 to conduct *de novo* SNP calling, and the results showed a comparable number of SNPs with the previous strategy (Eaton, 2014). Hence, we used only the SNPs generated using the first strategy for phylogenetic analyses. The SNPs were used to reconstruct the nuclear phylogenetic tree in IQ-TREE (Kalyaanamoorthy *et al*., 2017) and support values were obtained with 1000 replications of ultrafast bootstrapping (Hoang *et al*., 2018).

### Ancestral character reconstruction

Pappus morphology of the sampled species was determined via observations on living plants and/or herbarium specimens (BM, BRNU, E, HIB, IBSC, IBK, K, KUN, KYO, P, PE, PR, PRC, TI and TNS), supplemented by literature descriptions. Five pappus types were identified in Asterinae: long pappus, short pappus, dimorphic pappus, double pappus and lacking pappus (Fig. 1). Given the substantially broader sampling of the plastid phylogenetic tree than the nuclear SNP tree and to obtain a better representativeness of the evolution of pappus morphology, the plastid tree was used as the framework for the ancestral state reconstruction of pappus states with inference conducted using the ML method in the R package phytools v.0.7-47 (Revell, 2012). Although some phylogenetic incongruences exist between the plastid and nuclear SNP trees, these did not significant influence the evolutionary pattern because the affected taxa generally have the same type of long pappus. The single rate mode ‘ER’ was applied in character state reconstruction. An alternative strategy for character coding was used to investigate the influence of coding strategy on the evolution of pappus morphology. Pappus morphology was divided into four independent characters: (1) presence of the outer pappus bristle in ray florets, (2) length of the inner pappus bristle in ray florets, (3) presence of the outer pappus bristle in disc florets and (4) length of the inner pappus bristle in disc florets.

## RESULTS

### Plastid phylogenetic relationship of Asterinae

The plastid ML trees reconstructed using partitioned models were similar to those obtained from the non-partitioned model, with support values for the same clade being very similar. In addition, the phylogenetic topologies of BI trees were similar to those of ML trees (Supplementary Data Figs S1 and S2). Therefore, the best plastid ML tree (−Lh = 33 312.33 ± 540.30) reconstructed using partitioned models in IQ-TREE is discussed in the context of Asterinae phylogeny (Figs 2 and 3). The ultrafast bootstrap values (UBS) calculated using IQ-TREE, rapid bootstrap values (RBS) calculated using RAxML and Bayesian posterior probabilities (PP) calculated using MrBayes of important branches are indicated on these trees. Our results showed that the monophyly of the tribe Astereae was strongly supported (Fig. 2; UBS = 100, RBS = 100 and PP = 1), and most nodes within the backbone of Astereae and Asterinae were well resolved (Fig. 2).

**Fig. 2.**
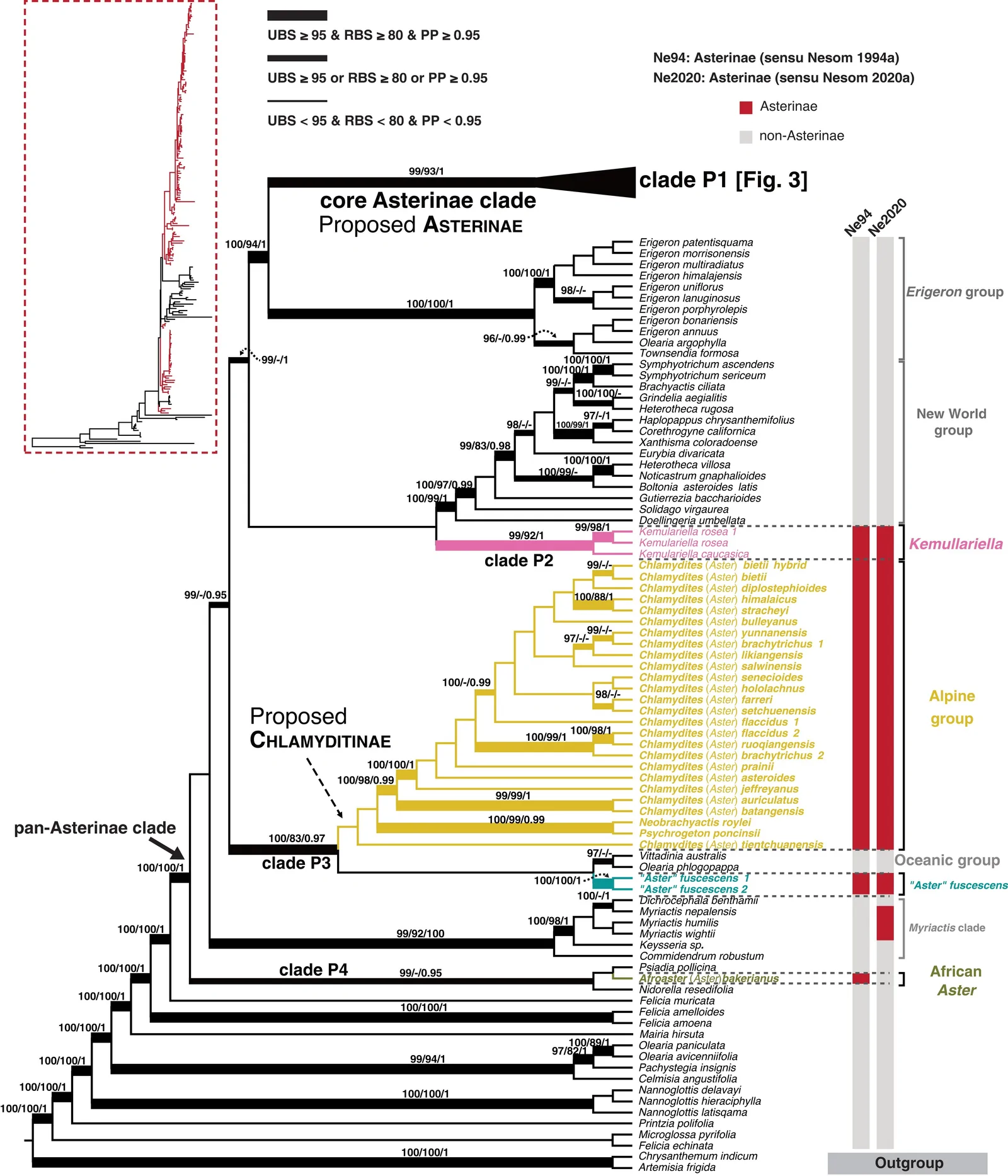
Plastid phylogenetic cladogram of Asterinae *s.l.* showing the relationships of four clades of Asterinae. The cladogram is from an ML tree derived from a concatenated matrix of five plastid spacer regions. Four clades recognized in traditional Asterinae are indicated as clades P1–P4. The previous classifications are shown on the right side and the meaning of the colours is shown at the top. Ne94: circumscription of Asterinae proposed in Nesom (1994*a*); Ne2020: circumscription of Asterinae proposed in Nesom (2020*a*). The width of the branch indicates support values of the corresponding clade and support values of important clades are shown on the branches. Support values on branches represent ultra-fast bootstrap (UBS), rapid bootstrap values (RBS) and Bayesian posterior probabilities (PP) respectively. Clades without support values correspond to UBS < 95, RBS < 80 and PP < 0.95.

**Fig. 3.**
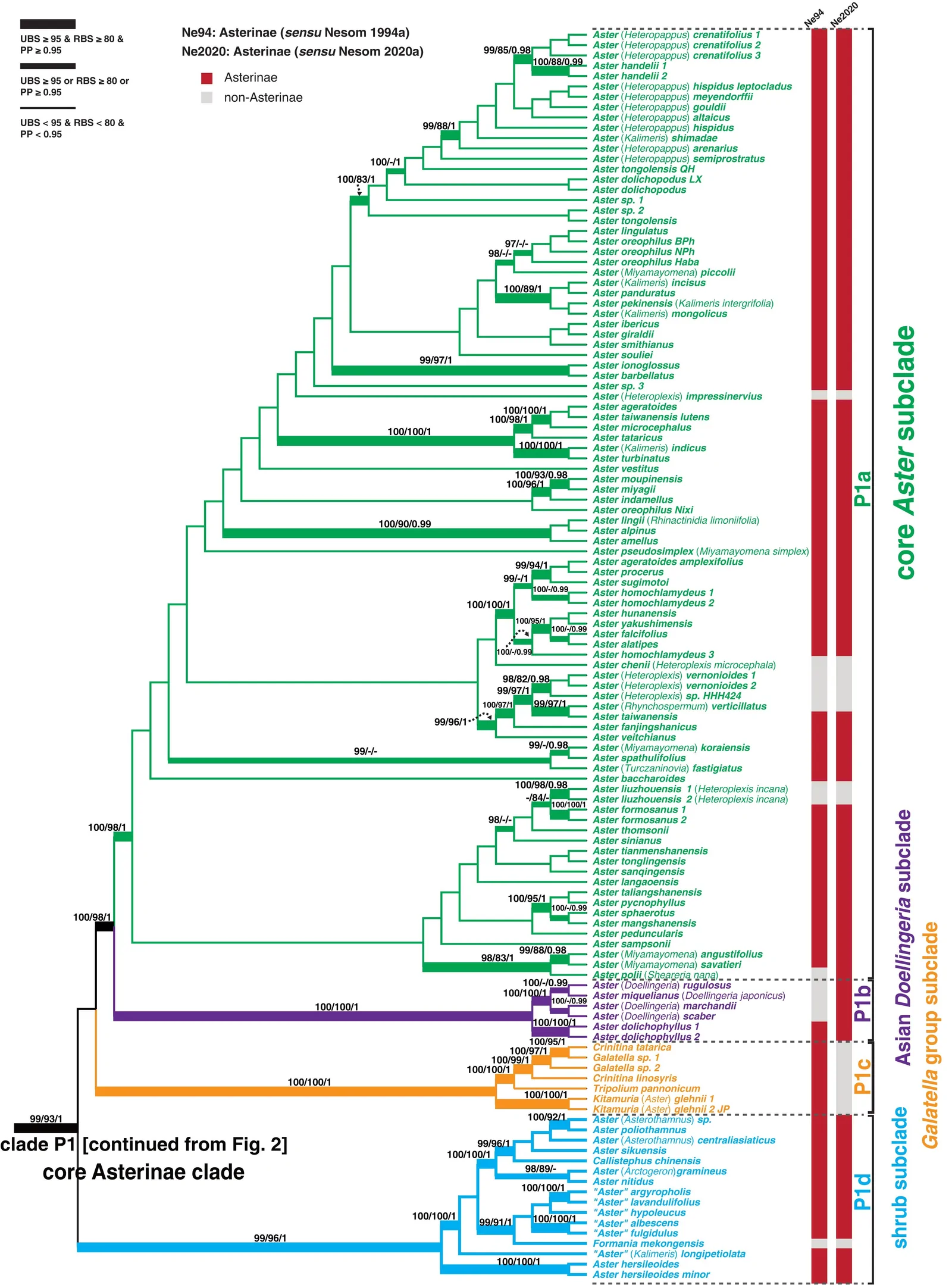
Plastid phylogenetic tree of the core Asterinae clade. The cladogram is an expanded view of Clade P1 (core Asterinae clade) from Fig. 2, continuing the framework from the preceding figure. Four subclades recognized in the core Asterinae clade are marked in different colours. The previous classifications are shown on the right side and the meaning of the colours is shown at the top. Ne94: circumscription of Asterinae proposed in Nesom (1994*a*); Ne2020: circumscription of Asterinae proposed in Nesom (2020*a*). The width of the branch indicates support values of the corresponding clade and support values of important clades are shown on the branches. Support values are shown on branches indicating ultra-fast bootstrap (UBS), rapid bootstrap values (RBS) and Bayesian posterior probabilities (PP) respectively; clades without support values indicate UBS < 95, RBS < 80 and PP < 0.95.

All members of Asterinae, along with ∼40 species from other subtribes or unplaced genera of Astereae, were resolved in the maximally supported pan-Asterinae clade (Fig. 2). Within this pan-Asterinae clade, the Asterinae species were further divided into four clades (clades P1–P4; Fig. 2): the core Asterinae clade (clade P1, Fig. 2; UBS = 99, RBS = 93 and PP = 1), the *Kemulariella* clade (clade P2, Fig. 2; UBS = 99, RBS = 92 and PP = 1), the alpine clade (clade P3, Fig. 2; UBS = 100, RBS = 83 and PP = 0.97) and the African clade (clade P4, Fig. 2; UBS = 99, RBS < 80 and PP = 0.95). Some species from other subtribes and unplaced genera of Astereae formed three distinct clades that intermixed with the four Asterinae clades and were nested within the pan-Asterinae clade. These include the *Myriactis* clade, the New World clade and the *Erigeron* group clade. Additionally, some non-Asterinae species were revealed nested within the Asterinae clades (clades P1, P3 and P4; Figs 2 and 3).

The core Asterinae clade, comprising 106 species sampled (clade P1; Figs 2 and 3), was strongly supported across all our analyses (UBS = 99, RBS = 93 and PP = 1). Three samples representing two *Kemulariella* species formed a strongly supported clade (clade P2, Fig. 2; UBS = 99, RBS = 92 and PP = 1) outside the core Asterinae. This clade was placed as sister to the New World clade, providing strong support for the monophyly of *Kemulariella*. Twenty-eight species belonging to *Aster* sect. *Alpigeni* (as circumscribed in *Flora of China*) have generally solitary heads with fully herbaceous phyllaries. These species, distributed in the alpine regions of the Hengduan Mountains and the Himalayas at elevations generally above 3000 m, were defined as the alpine *Aster*. It included the species numbered 95–122 in the treatment of the genus *Aster* in *Flora of China* (Chen *et al*., 2011). Twenty-three samples of the alpine *Aster*, three samples of *A. auriculatus* and *A. fuscescens*, and four species of *Neobrachyactis*, *Psychrogeton*, *Vittadinia* and *Olearia* formed the alpine clade (clade P3, Fig. 2; UBS = 100, RBS = 83 and PP = 0.97). Additionally, *Aster bakerianus* and two non-Asterinae genera, *Psiadia* and *Nidorella* – all distributed in Africa – formed the well-supported African clade (clade P4, Fig. 2; UBS = 99, RBS < 80 and PP = 0.95).

Our phylogenetic analyses indicated that the core Asterinae clade contained four strongly supported subclades, i.e. the core *Aster* subclade (subclade P1a in Fig. 3; UBS = 100, RBS = 98 and PP = 1), the Asian ‘*Doellingeria*’ subclade (subclade P1b, Fig. 3; UBS = 100, RBS = 100 and PP = 1), the *Galatella* group subclade (subclade P1c, Fig. 3; UBS = 100, RBS = 100 and PP = 1) and the shrub subclade (subclade P1d, Fig. 3; UBS = 99, RBS = 96 and PP = 1). The core *Aster* subclade and the Asian ‘*Doellingeria*’ subclade formed a strongly supported clade that is sister to the *Galatella* group subclade. The shrub subclade was identified as the earliest-diverging lineage within the core Asterinae clade. The core *Aster* subclade (clade P1a, Fig. 3; UBS = 100, RBS = 98 and PP = 1), containing most members of *Aster*, including the type species *A. amellus*, along with eight closely related genera, was the largest subclade of the core Asterinae clade (Fig. 3). The Asian ‘*Doellingeria*’ subclade (subclade P1b, Fig. 3; UBS = 100, RBS = 100 and PP = 1) contained one species of *Aster* and four species of ‘*Doellingeria*’ distributed in East Asia. The *Galatella* group subclade (Fig. 3; UBS = 100, RBS = 100 and PP = 1) consisted of all three genera of *Galatella* group and *Aster glehnii*. The shrub subclade (Fig. 3; UBS = 99, RBS = 96 and PP = 1) contained two shrubby genera (*Asterothamnus* and *Formania*) and multiple shrubby species of *Aster*. Notably, three herbaceous groups, *Arctogeron*, *Callistephus* and *Kalimeris longipetiolata*, were nested within this predominantly woody subclade.

### Nuclear phylogeny of Asterinae

In total, 6 614 469 SNPs were obtained from the ddRADseq datasets of 120 species and one variety of the tribe Astereae, and the number of retained SNPs filtered using different coverages of species is provided in Supplementary Data Table S4. To optimize species coverage and phylogenetic resolution, we utilized a filtered set of 130 503 SNPs shared by at least 20 taxa (designated as the S20 set; Table S4) for the nuclear phylogeny of Astereae; that is, those sites that are SNPs that were not missing in more than 20 % species were retained for subsequent analysis. The missing rate of this dataset is about 60 %. The best ML tree (−Lh = 1 606 271.12, Fig. 4) reconstructed using this dataset strongly supported the monophyly of Astereae, and most internal nodes within the tribe were well resolved with bootstrap values ≥95 %. Nuclear phylogenetic reconstruction revealed the non-monophyly of Asterinae, with members distributed across seven clades (Fig. 4). Most species of Asterinae were strongly supported to form a monophyletic group (BS = 97) and contained four strongly supported clades (N1–N4), while three additional clades (N5–N7) exhibited divergent phylogenetic affiliations. The largest clade, N1 (BS = 97), included 54 species, encompassing the majority of *Aster*, four Asian ‘*Doellingeria*’ species, *Kalimeris integrifolia*, and all sampled species of *Arctogeron*, *Asterothamnus*, *Heteropappus*, *Heteroplexis*, *Miyamayomena*, *Rhynchospermum*, *Sheareria* and *Turczaninowia*. Clade N2 (BS = 100) contained *Callistephus chinensis* and *Kemulariella rosea*, whereas Clade N3 (BS = 100) contained *Neobrachyactis* and *Pyschrogeton*. Clade N4 (BS = 100) comprised all alpine *Aster* species, *A. auriculatus*, *A. fuscescens*, *A. veitchianus*, *A. pycnophilus*, *Kalimeris longipetiolata*, *Formania mekongensis*, and three shrub *Aster* species, i.e. *A. lavandulifolius*, *A. argyropholis* and *A. fulgidulus*. Outside this core radiation, Clade N5 (BS = 100) emerged as a *Galatella*-associated lineage sister to *Bellis*, including two *Galatella* species, *Crinitina linosyris*, *Tripolium pannonicum* and *A. glehnii*. The African *Aster bakerianus* and *Kemulariella caucasica* were resolved as Clade N6 and Clade N7 (both BS = 100 for the placements), respectively. These two species were grouped together with African Astereae species and *Nannoglottis* (BS = 100), being geographically distant from other Asterinae groups.

**Fig. 4.**
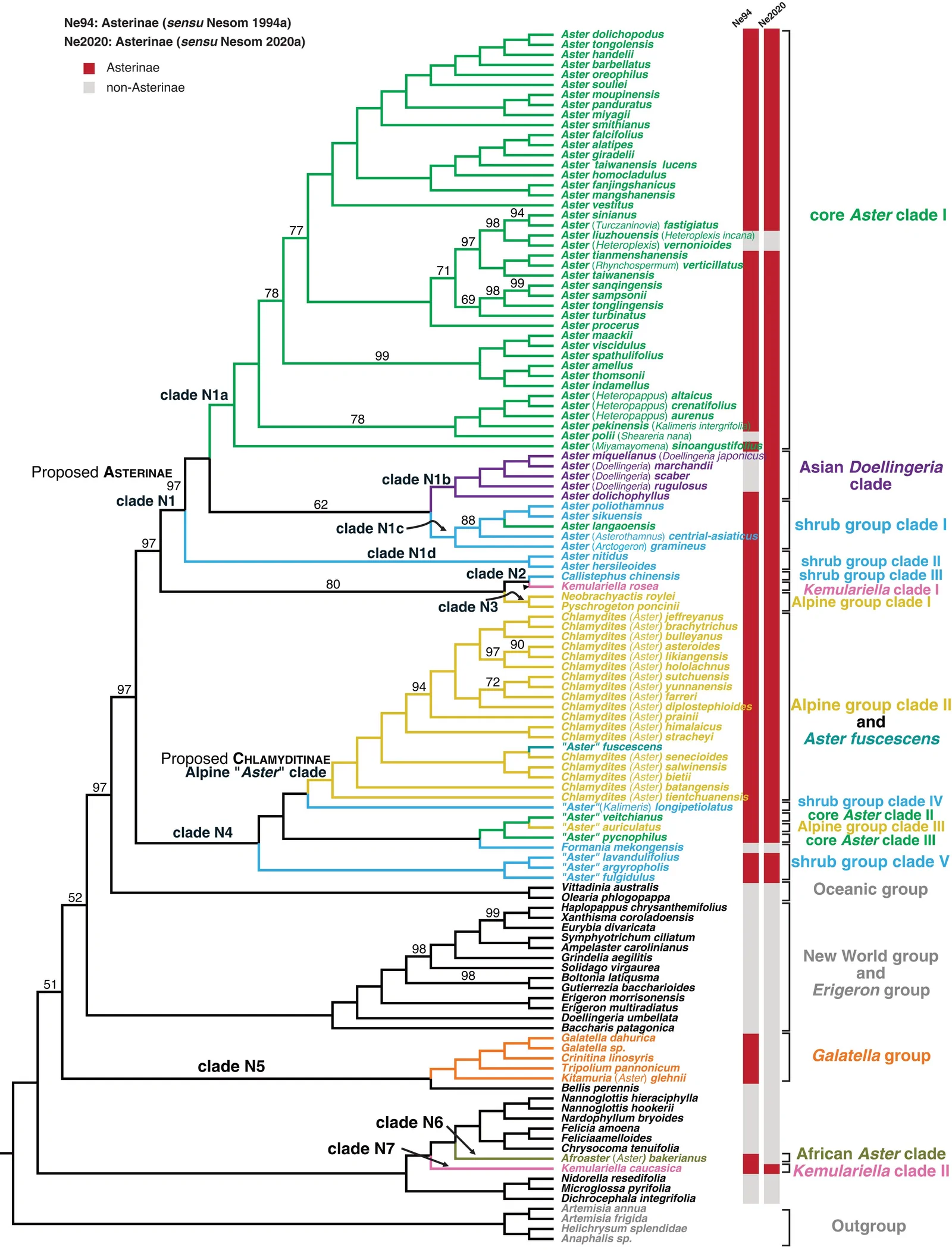
Nuclear phylogentic tree of Asterinae reconstructed using nuclear SNPs. The S20 SNP dataset was used to reconstruct the phylogenetic tree using the maximum-likelihood (ML) method in RAxML. Bootstrap support values (BS) are indicated on the branches unless the branches have BS of 100. The clades and subclades defined in the plastid phylogenetic trees are indicated on the right side of the tree and using branch colours.

Within Clade N1, four maximally supported subclades (BS = 100) N1a–N1d were revealed (Fig. 5). Clade N1a, the largest one, contained 41 species and one variety, including 31 *Aster* species (plus one variety), *Heteropappus*, one *Kalimeris* species, *Miyamayomena* and *Turczaninowia* of Asterinae, and three non-Asterinae genera, *Heteroplexis*, *Rhynchospermum* and *Sheareria*. Clade N1b comprised four Asian ‘*Doellingeria*’ and *A. dolichophyllus*, while Clade N1c consisted of three subshrub *Aster* species, *Asterothamnus* and *Arctogeron*. Clade N1d, identified as the earliest-diverging lineage within N1, consisted exclusively of two species of *Aster* ser. *Hersileoides* [*A. hersileoides* and *A. nitidus* (Ling, 1985)]. Clade N1b and Clade N1c were resolved as sister groups with moderate support (BS = 62).

**Fig. 5.**
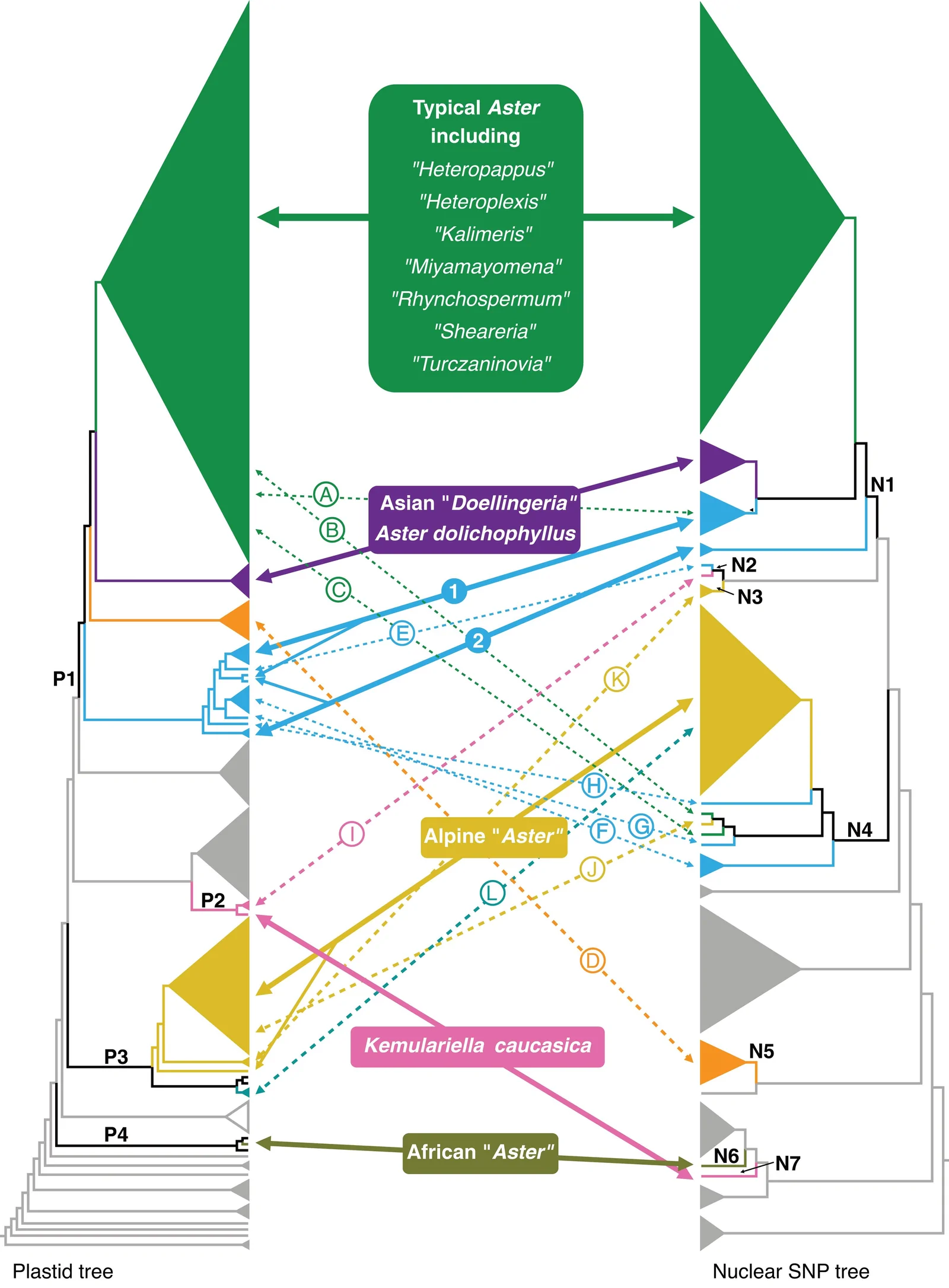
Comparison of the plastid (left) and nuclear (right) phylogenetic trees of Asterinae. Groups that are consistently supported by both plastid and nuclear phylogenetic trees are indicated using solid arrows between the two trees. The names of taxa are shown on corresponding arrows (1: *Asterothamnus*, *Aster poliothamnus*, *A. sikuensis* and *Arctogeron*; 2: *Aster hersileoides* and *A. nitidus*). Groups that have discordant placements in plastid and nuclear phylogenetic trees are indicated using dashed arrows between the two trees (A: *Aster langaoensis*; B: ‘*A.*’ *veitchianus*; C: ‘*A.*’ *pycnophilus*; D: *Galatella* group and *Kitamura* (*Aster*) *glehnii*; E: *Callistephus*; F: *A.* ser. *Albescentes*, i.e. *A. albescens* and close relatives; G: *Formania*; H: ‘*A.*’ (*Kalimeris*) *longipetiolata*; I: *Kemulariella rosea*; J: ‘*A.*’ *auriculatus*; K: *Neobrachyactis* and *Psychrogeton*; and L: ‘*A.*’ *fuscescence*). Clades recognized in the plastid and nuclear SNP trees are indicated with their names on corresponding branches.

### Concordance and discordance between plastid and nuclear SNP phylogenies

Our analyses revealed both congruent and conflicting phylogenetic signals between the plastid and nuclear SNP datasets (Fig. 5). Seven major clades of Asterinae received consistent support across both phylogenies (Fig. 5). The plastid-defined P1 (the core Asterinae clade), encompassing most Asterinae genera and species, corresponded strongly to nuclear Clade N1. Notably, the plastid-derived core *Aster* lineage (P1a), comprising typical *Aster* species (e.g. *A. amellus* L., the type species of the genus) and allied genera (*Heteropappus*, *Heteroplexis*, *Kalimeris*, *Miyamayomena*, *Rhynchospermum*, *Sheareria* and *Turczaninowia*), showed nuclear counterpart N1a with maximal support (BS = 100), though excluding *A. veitchianus*, *A. pycnophilus* and *A. langaoensis* (Figs 4 and 5). Asian ‘*Doellingeria*’ taxa and *A. dolichophyllus* formed congruent clades (P1b/N1b; BS = 100) in both trees (Figs 3–5). Similarly, some shrubby species of *Aster* (*A. poliothamnus* and *A. sikuensis*, *Asterothamnus* and *Arctogeron*) were clustered consistently in plastid P1d and nuclear Clade N1c (‘consistent clade 1’ in Fig. 5). Additionally, our plastid and nuclear analyses supported that *Aster hersileoides* and *A. nitidus* have close relationships (Clade N1d), although they were not sister groups within Clade P1d in the plastid tree (consistent clade 2; Fig. 5). Furthermore, the alpine *Aster* species were consistently placed within clades distant from other Asterinae groups in both plastid and nuclear phylogenetic trees (P3 in plastid trees, N4 in nuclear trees; Figs 2, 4 and 5). These alpine *Aster* species formed a well-supported clade in both analyses, with two notable exceptions: (1) *A. tientchuanensis* showed weak affinity (BS < 50) to the core alpine clade in plastid phylogenies, and (2) *A. fuscescens* was included in the alpine clade in the nuclear analysis (Figs 2, 4 and 5). *Kemullariella caucasica*, the type species of the genus, and the African *A. bakerianus* were consistently resolved distantly from most Asterinae groups in both plastid and nuclear SNP trees (Figs 2, 4 and 5). The agreement of the placements of these clades suggested that most relationships of Astereae were consistent between plastid and nuclear phylogenetic analyses.

Our analysis also revealed 12 critical instances of cytonuclear discordance (designated as discordant groups A–L; Fig. 5). These discordant groups exhibit significantly different phylogenetic placements across the trees reconstructed from the plastid and nuclear SNP datasets. *Aster langaoensis* (discordant group A; Fig. 5), a subshrub species, was placed within the core *Aster* clade (Clade P1a) in the plastid tree but was placed outside of the core *Aster* clade (Clade N1a) and clustered with other subshrub species (*A. poliothamnus*, *A. sikuensis* and *Asterothamnus*) in the nuclear SNP tree (Figs 3–5). *Aster veitchianus* and *A. pycnophilus* (discordant groups B and C; Fig. 5) were resolved within the core *Aster* clade (Clade P1a) in the plastid tree but were shown to be closely related to *A. auriculatus* and the alpine *Aster* species in the nuclear SNP tree (Figs 3–5). The *Galatella* groups and *A. glehnii* (discordant group D; Fig. 5) were resolved as a strongly supported clade within the core Asterinae clade in the plastid tree (Clade P1c; Fig. 3). Their monophyly was also maximally supported in the nuclear SNP tree (Clade N5; Fig. 4), whereas they were shown to be closely related to *Bellis* rather than the core Asterinae groups in the nuclear SNP tree (Fig. 4). *Callistephus chinensis* (discordant group E; Fig. 5) was grouped together with the subshrub species of *Aster* and *Asterothamnus* in the plastid tree (within Clade P1d; Fig. 3), whereas it was shown to be the sister group of *Kemulariella rosea* and distant from the subshrub species of *Aster* (Clade N2; Fig. 4). Species of *Aster* ser. *Albescentes* (*sensu*Ling, 1985) (discordant group F; Fig. 5) and *Formania* (discordant group G; Fig. 5) are shrubs and were resolved within the shrub clade of core Asterinae in the plastid tree, closely related to the subshrub species of *Aster* mentioned above (Clade P1d; Fig. 3). By contrasting, these shrub species were shown to closely related to the alpine *Aster* in the nuclear SNP phylogenetic tree (Clade N4; Figs 4 and 5). Additionally, the herbaceous *Kalimeris longipetiolata* (discordant group H; Fig. 5) was shown to be closely related to the subshrubs and shrubs mentioned above in the plastid tree (Clade P1d; Fig. 3). However, it was resolved as the sister group of the exclusively herbaceous alpine *Aster* in the nuclear SNP tree (Clade N4l; Figs 4 and 5). *Kemulariella rosea* (discordant group I; Fig. 5) was sister to *K. caucasica* in the plastid tree (Clade P2; Figs 2 and 5) with BS > 92, but grouped with *Callistephus chinensis* in the nuclear SNP tree (clade N2; Figs 4 and 5) with maximal support. *Aster auriculatus* (discordant group J; Fig. 5) was nested within the alpine *Aster* species in the plastid tree (Fig. 2) and was grouped together with *A. veitchianus* and *A. pycnophilus* but still had close relationships with the alpine *Aster* (clades P3 and N4; Figs 3–5). Two alpine genera, *Neobrachyactis* and *Psychrogeton* (discordant group K; Fig. 5), grouped together with the alpine *Aster* species in the plastid tree but were resolved as the sister group of *Kemulariella rosea* plus *Callistephus chinensis* with BS of 80 in the nuclear SNP tree (clades P3 and N3; Figs 2, 4 and 5). Moreover, the plastid tree placed *A. fuscescens* (discordant group L; Fig. 5) outside of the alpine *Aster* clade (Clade P3; Fig. 3) but the nuclear SNP tree resolved it within the alpine *Aster* clade (Clade N4; Fig. 4) with maximal support (Figs 2, 4 and 5). In summary, our study revealed significant nuclear–plastid discordance for some Asterinae genera and species, despite most groups showing consistent placements in both plastid and nuclear SNP trees. Given the complex evolutionary histories of these groups, the taxonomic treatments made in this study (see below) focused only on the seven clades that were consistently supported by phylogenetic trees with different inheritance backgrounds.

### Evolutionary dynamics of pappus morphology in Asterinae

The ancestral reconstruction of pappus morphology revealed that a long pappus was dominant in Asterinae. The common ancestor of the pan-Asterinae clade had a long pappus, and both the ancestors of the African clade and the core Asterinae clade also had a long pappus (Fig. 6). In contrast, the common ancestors of both the alpine clade and the *Kemulariella* clade had a double pappus that was dominant in these two clades. In the core Asterinae clade, each of the four subclades had a common ancestor with a long pappus. The short pappus independently originated at least 19 times in the pan-Asterinae clade, with at least 14 instances involving a transition from long pappus to short pappus (pappus length reduction in all achenes). The transition from double pappus to short pappus was detected four times and only detected in the alpine clade. Notably, the short pappus in *Kalimeris shimadai* probably derived from the dimorphic pappus, differing from other core Asterinae species, and indicating the different origins of the short pappus. The dimorphic pappus (pappus length reduction in achenes of ray florets) originated three times: twice in the core Asterinae clade and once in Conyzinae, all transitioning from the long pappus (Fig. 6). The largest clade with dimorphic pappus mainly comprised *Heteropappus* species. The double pappus evolved separately three times from the long pappus in the alpine clade, the *Kemulariella* clade and the core Asterinae clade. It was dominant in the former two clades and found only in one species of the core Asterinae clade. Our study also identified five instances of pappus loss: four in the core Asterinae clade and one in the *Myriactis* clade (Fig. 6). In addition, as pappus morphology is a complex character, we subdivided it into four independent states to explore the effects of different coding strategies on ancestral state reconstruction (Supplementary Data Fig. S3). Our results strongly supported the pattern recovered in the previous analysis, indicating that alternative character coding schemes do not alter the evolutionary pattern of pappus morphology.

**Fig. 6.**
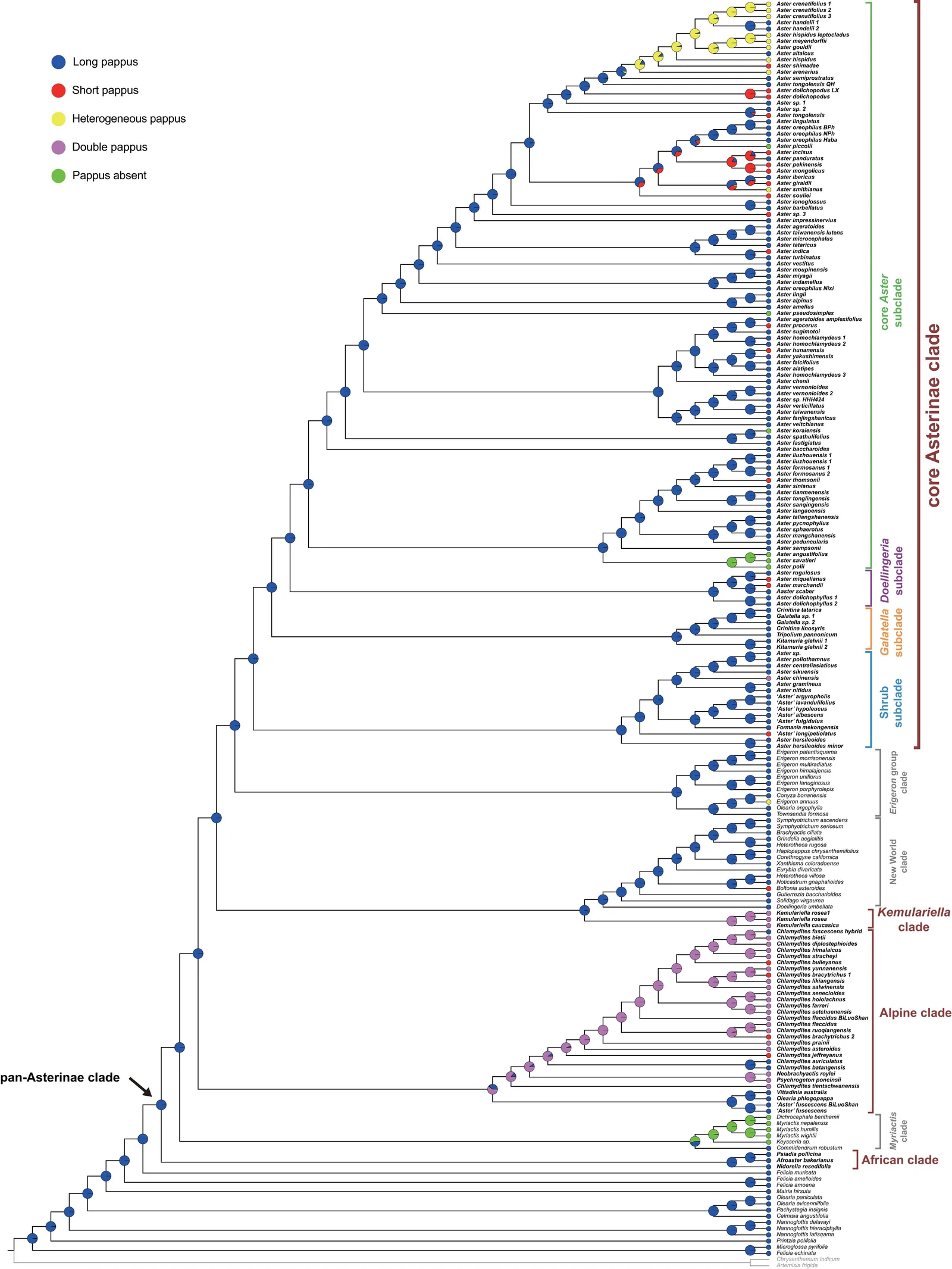
Ancestral state reconstruction of the pappus on the basis of the plastid phylogenetic tree showing convergent evolution of different types of pappus. Colours at tips show the pappus type of the corresponding taxa; pie charts at nodes show the probabilities of the estimated pappus types. Clades recognized in this study are shown on the right side.

## DISCUSSION

### New phylogeny and re-delimitation of Asterinae

With comprehensive sampling across all recognized genera and broad species representation, both plastid and nuclear SNP phylogenies consistently demonstrate the non-monophyly of Asterinae as previously (Nesom, 1994*a*; Nesom and Robinson, 2007) and recently (Nesom, 2020*a*) circumscribed. Both plastid and nuclear phylogenies revealed the need for re-delimitation subtribe Asterinae (Figs 2–4). Most genera and about two-thirds of the species previously assigned to Asterinae (Ling, 1985; Nesom, 1994*a*, *b*; Nesom and Robinson, 2007; Chen *et al*., 2011) were placed in a well-supported clade in both datasets (clades P1 and N1; Figs. 3 and 4, respectively). This includes *Aster amellus* – the type species of both the genus *Aster* and subtribe Asterinae – which was consistently embedded in these clades (Figs 3 and 4). Also, these two clades had nearly congruent taxonomic composition except that a few species were excluded from Clade N1 in the nuclear phylogeny.

*Turczaninowia*, previously classified within *Aster* by Nesom (1994*b*), was resolved within Asterinae. Notably, *Heteroplexis*, a shrubby genus endemic to karst areas of southwestern China, was previously treated as a member of the *Erigeron–Conyza* group (Zhang and Bremer, 1993) or considered as taxonomically unplaced (Nesom and Robinson, 2007) based on morphological characters. Also, *Heteroplexis* was placed in the *Psiadia* group of the subtribe Grangeinae, a group mainly distributed in Africa, in the recent classification of the tribe (Nesom, 2020*a*). However, our results revealed that *Heteroplexis* species were scattered in Asterinae, marking the first molecular evidence for its non-monophyly. The type species of *Doellingeria*, *D. umbellatum*, was resolved within a New World Astereae lineage (the New World clade; Fig. 2), whereas the four Asian ‘*Doellingeria*’ species clustered and nested in Asterinae, supporting recent analysis recognizing true *Doellingeria* species as a North American group (Allen *et al*., 2019). *Sheareria* and *Rhynchospermum*, two monotypic genera once classified in Lagenophorinae (Nesom and Robinson, 2007) based on their epappose achenes (or with caducous pappus in *Rhynchospermum*), were in Asterinae, aligning with previous evidence linking *Rhynchospermum* to *Aster* (Brouillet *et al*., 2009). Additionally, *Formania*, a monotypic shrubby genus endemic to dry warm valleys of southwestern China, was originally assigned to Anthemideae due to its pinnatilobate leaves (Shih and Fu, 1983). Recently, this genus was transferred to Astereae (Fu *et al*., 2016; Chen *et al*., 2011) and treated as a monotypic subtribe Formaniinae (Nesom, 2020*a*) based mainly on the morphology and limited phylogenetic data. Our phylogeny supported its membership in Astereae and further refines its position within Asterinae.

The redefined Asterinae includes all *Aster* species (including *Turczaninovia*) except African *Aster* species (*Afroaster*), the alpine *Aster*, *A. auriculatus*, *A. fuscescense*, *A. veitchianus*, *A. pycnophilus* and members of *A.* ser. *Albescentes* (*sensu*Ling, 1985). It retains all species of *Arctogeron*, *Asterothamnus*, *Heteropappus* and *Miyamayomena* from traditional Asterinae circumscriptions of Asterinae (Nesom, 1994*a*, *b*; Nesom and Robinson, 2007). Additionally, it incorporates all species of *Heteroplexis*, *Rhynchospermum* and *Sheareria*, and the Asian ‘*Doellingeria*’ species, which were previously excluded from Asterinae (Nesom and Robinson, 2007). Key potential morphological synapomorphies for this clade include: obviously one-seriate ray florets, narrowly campanulate involucres, base-hardened phyllaries, and usually flat and 2–3-ribbed achenes (Fig. 7A–L). While *Rhinactinidia* was not sampled in the nuclear analyses, morphological evidence suggests affinity with the newly defined Asterinae as a part of the typical genus *Aster*. In contrast, *Myriactis* (*Myriactis* clade; Fig. 2) – recently classified in Asterinae (Nesom, 2020*a*) but excluded from the core Asterinae clade in our plastid phylogeny – probably falls outside the newly defined Asterinae based on morphological and plastid phylogenetic evidence.

**Fig. 7.**
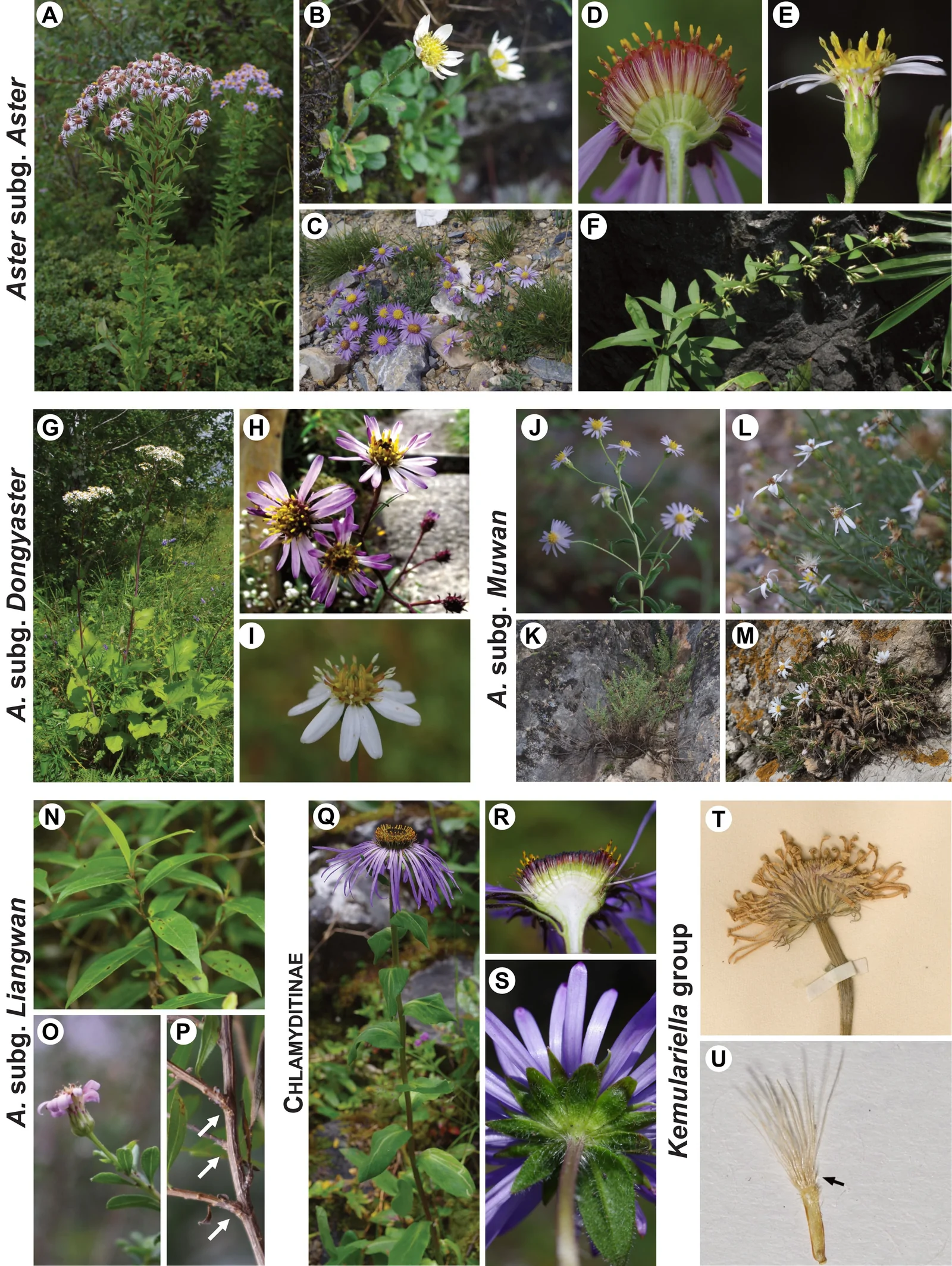
Diagnostic characters and representative species of newly defined Asterinae, *Kemulariella* group and Chlamyditinae. (A) *Aster vestitus* with corymbiform capitulescence; (B) *Aster fanjingshanicus*; (C) *Aster semiprostratus* (*Heteropappus semiprostratus*); (D) cross-section of the capitulum of *A. vestitus* showing long and single-series pappus with long bristles; (E) a capitulum of *A. tonglingensis* showing imbricate phyllaries arranged in four series and with hardened base; (F) *Aster chenii* (*Heteroplexis microcephala*); (G) *Aster scaber* (*Doellingeria scabra*); (H) capitula of *A. dolichophyllus* showing long lobes of disc corolla; (I) a capitulum of *A. rugulosus* showing long lobes of disc corolla; (J) capitula of *A. poliothamnus*; (K) many branches growing from rhizomes of *A. poliothamnus*; (L) *A. centraliasiaticus* (*Asterothamnus centraliasiaticus*); (M) *Aster gramineus* (*Arctogeron gramineum*) showing branched rhizomes with rosette leaves and solitary capitula; (N) glabrous leaves of *Aster nitidus*; (O) a capitulum of *A. hersileoides*; (P) branches of *A. hersileoides* with persistent scales at the bases (white arrows); (Q) *Chlamydites bietii* showing solitary capitulum; (R) cross-section of the capitulum of *C. yunnanensis* showing double-series pappus with outer layer composed of scales and inner layer composed of bristles; (S) a capitulum of *C. brachytrichus* showing fully herbaceous phyllaries; (T) a capitulum of *Kemulariella caucasica* showing fully herbaceous phyllaries; (U) a cypsela of *K. caucasica* showing double-series pappus. A–P belong to *Aster* species (Asterinae) with A–F from species of *A.* subg. *Aster*, G–I from species of *A.* subg. *Dongyaster*, J–M from species of *A.* subg. *Muwan* and N–P from species of *A.* subg. *Liangwan*. Q–S belong to *Chlamydites* species (Chlamyditinae). T and U belong to *Kemulariella caucasica* (*Kemulariella* group). Photos F and M were taken by Haihua Hu; N and O by Zhixi Fu; others by Guojin Zhang.

Cytonuclear discordances between the two phylogenies were detected in this study. The nuclear SNP phylogeny resolved *Aster albescens* and its closely related shrub species, *Kalimeris longipetiolata*, and *Formania* within Clade N4 (Fig. 4), contrasting with their placement in Clade P1d based on the plastid result (Fig. 3). Similarly, *Callistephus* was placed in Clade N2 instead of Clade N1 in the nuclear phylogeny (Fig. 4), but clustered in Clade P1d in the plastid phylogeny (Fig. 3). Two core *Aster* species (*A. veitchianus* and *A. pycnophilus*) nested in plastid Clade P1d (Fig. 3), instead allied with *A. auriculatus* in nuclear Clade N4 (Fig. 4). Additionally, *A. langaoensis* grouped within the plastid core *Aster* clade (Clade P1d; Fig. 3) but clustered with other subshrub species of *Aster* in the nuclear phylogeny (Clade N1c; Fig. 4). These cytonuclear discordances (Fig. 5) may reflect historical hybridization, though other processes (e.g. incomplete lineage sorting) could not be ruled out, suggesting further research on hybridization using genomic data is needed for a comprehensive understanding of the evolution of this subtribe.

### Generic taxonomy of Asterinae: re-circumscription of *Aster* and its allies

Within the recircumscribed Asterinae, the previously defined genus *Aster* was resolved as polyphyletic, with *Arctogeron*, *Asterothamnus*, Asian ‘*Doellingeria*’, *Heteropappus*, *Heteroplexis*, *Kalimeris* (except *K. longipetiolata*), *Miyamayomena*, *Rhynchospermum*, *Sheareria* and *Turczaninovia* nested within it (Figs 3 and 4). These findings partially align with the treatment in the *Flora of China* (Chen *et al*., 2011) and a previous study (Li *et al*., 2012). Notably, *A. amellus* and *A. indamellus*, both belonging to *Aster s.s.* proposed recently (*sensu*Nesom, 2020*b*), failed to form a monophyletic group in plastid and nuclear phylogenetic trees, suggesting that *Aster s.s.* is also not monophyletic (Fig. 3 and 4). Most of the species placed within this clade were previously placed within *Aster s.l.* and shared the following three diagnostic morphological characters: corymbiform capitulescence, phyllaries with an herbaceous apex and hardened base, and single-row ray florets. Based on comprehensive morphological and phylogenetic evidence, we propose a revised generic circumscription of *Aster* to include all species within the newly defined Asterinae.

### Infrageneric taxonomy of *Aster*

Four strongly supported subgeneric clades (N1a–N1d; Fig. 4) were resolved within the recircumscribed *Aster* (Fig. 4). Clade N1a was also supported in the plastid phylogeny with strong support except for *A. langaoensis*, *A. veitchianus* and *A. pycnophilus* mentioned above. This clade is characterized by herbaceous habit, loose corymbiform capitulescence, >20 ray florets per capitulum, disc floret lobes <1/3 corolla length and uniseriate pappus.

Four Asian ‘*Doellingeria*’ species and *A. dolichophyllus* formed a clade consistently strongly supported in both nuclear and plastid phylogenies (clades N1b and P1b; Figs 3 and 4). This clade is characterized by herbaceous habit, loose corymbiform capitulescence, <15 ray florets per capitulum, disc florets lobes >1/2 corolla length and uniseriate pappus.

Three subshrubby *Aster* species alongside one species from *Asterothamnus* and *Arctogeron* formed a strongly supported clade (N1c) in the nuclear SNP phylogeny (Fig. 4). All these species are subshrubs except *Arctogeron*, which is herbaceous but has woody, branched rhizomes. While plastid data placed these species within a broader shrub clade (P1d; Fig. 3), they exhibit distinct biogeographical and morphological characters: distributed in the region north of the Daba Mountains of China (vs. south of the Daba Mountains of China for other species in Clade P1d in the plastid phylogeny); and multi-stemmed growth from rhizomes (except *Arctogeron* with solitary scapes on well-branched rhizomes) contrasting with without many branches on a single or a few main stems for other shrub species in clade P1d in the plastid phylogeny.

*Aster nitidus* and *A. hersileoides* (assigned to *A.* ser. *Hersileoides sensu*Ling, 1985) formed a maximally supported clade (N1d; Fig. 4) in the nuclear phylogeny. These two southwestern Chinese shrubs species differ from other shrub *Aster* species by longer ray florets, glabrous leaves and blossom in spring to early summer (vs. hairy leaves and blossom in later summer to autumn).

Based on the phylogenetic, morphological and distribution evidence, we propose formal recognition of these four nuclear-derived *Aster* clades (N1a–N1d) as four subgenera within *Aster*.

### Phylogenetic position of *Kemulariella*

*Kemulariella*, a genus historically segregated from *Aster* sect. *Caucasica* (Tamamschjan, 1959) based on its double pappus (Tamamschjan, 1959; Nesom and Robinson, 2007), comprises six species endemic to the Caucasus (Tamamschjan, 1959; Grierson, 1975; Nesom and Robinson, 2007; Firat, 2016). Our phylogenetic analyses included two *Kemulariella* species (*K. caucasica* and *K. rosea*), establishing their molecular phylogenetic placement for the first time. Plastid data strongly supported the monophyly of these species (Clade P2; Fig. 2), forming a sister relationship to the New World lineages rather than the core Asterinae clade (Fig. 2). This contrasted sharply with nuclear phylogenetic results, where *Kemulariella* was not a monophyletic genus, *K. caucasica* (the type species) was resolved in an isolated position outside Asterinae (Clade N7; Fig. 4), while *K. rosea* was resolved as sister to *Callistephus chinensis* (Clade N2; Fig. 4) in a lineage sister to *Neobrachyactis* and *Psychrogeton*. These results showed incongruent phylogenetic positions of the genus and suggested that *K. rosea* might have a hybrid origin. The unique phylogenetic position, morphological characters, including double pappus, acute phyllary tips and slightly compressed achenes (Fig. 6M, N), coupled with its endemic Caucasus distribution of *Kemulariella caucasica* supported its recognition as representing an independent lineage named ‘*Kemulariella* group’. Although the evidence above strongly supports recognition of this clade at subtribe rank (as subtribe Kemulariellinae), only two of the six recognized species in *Kemulariella* were sampled here. To avoid potential nomenclatural instability, we adopted a conservative taxonomic approach and refrained from a formal establishment of the subtribe here, pending broader taxon sampling in future phylogenetic analyses. We encourage collaborative efforts to expand taxon sampling to test this recognition and clarify its circumscription. The aberrant placements of *K. rosea* in nuclear SNP data, combined with incomplete sampling of the genus (4/6 species not analysed), precludes definitive conclusions. This study resolves long-standing uncertainties regarding the phylogenetic position of *Kemulariella* first raised by Tamamschjan (1959), demonstrating that its traditional separation from *Aster* based on pappus morphology corresponds to deeper evolutionary divergence than previously recognized. Nevertheless, the complex history implied by cytonuclear discordance underscores the need for integrative taxonomic approaches when delimiting lineages in rapidly diversifying groups like Astereae.

### Phylogenetic placement and classification of the alpine *Aster* species

Plastid phylogenetic analysis of 23 samples representing 20 alpine *Aster* species revealed that 22 samples together with *Aster auriculatus* formed a strongly supported clade within Clade P3 (UBS = 100, RBS = 98 and PP = 0.99; Fig. 2). Although *Aster auriculatus* has been traditionally treated as a typical *Aster* species, it shares similar characters and distribution with that of the alpine *Aster* and was resolved as sister to *A. batangensis* with strong support in our plastid phylogeny. This alpine *Aster* clade was sister to two other alpine genera (*Neobrachyactis* and *Psychrogeton*). Additionally, *A. tientchuanensis*, an alpine *Aster*, was resolved as sister to the clade containing the other alpine asters, *Neobrachyactis* and *Psychrogeton*. *Neobrachyactis* was far from typical *Brachyactis* (a synonym of *Symphyotrichum*) in our plastid phylogeny (the New World clade; Fig. 2), supporting the recognition of ‘para-*Brachyactis*’ (Nesom, 1994*b*) and recent separation of *Neobrachyactis* from *Brachyactis* (Chen and Brouillet, 2011). Additionally, *A. fuscescens*, also traditionally treated as a typical *Aster* species and distributed in the alpine region of the Hengduan Mountains, was shown to have close relationships with the alpine *Aster* species (Fig. 2), challenging its traditional taxonomic placement. Our nuclear phylogeny incorporated 18 alpine *Aster* species, which together with *A. fuscescens* formed a maximally supported clade (within N4; Fig. 4). *Aster tientchuanensis* occupied the basal position within this clade. Intriguingly, *Kalimeris longipetiolata*, a species phylogenetically allied with the shrub *Aster* in the plastid phylogenetic tree (Clade P1d; Fig. 3), was resolved as sister to the alpine *Aster* in our nuclear phylogeny (Clade N4; Fig. 4). This species, characterized by solitary heads and fully herbaceous phyllaries, exhibits geographical proximity to *A. tientchuanensis* in the eastern side of the Qionglai Mountain range, aligning its nuclear phylogenetic placement with morphological and distributional evidence. The significantly different placements of *K. longipetiolata* in the plastid and nuclear phylogenetic trees suggested that the species might originate from a hybridization event between alpine asters and a species related to shrub asters. Three *Aster* species (*A. veitchianus*, *A. pycnophilus* and *A. auriculatus*) formed a maximally supported group in the nuclear phylogeny (Fig. 4), with their diagnostic auriculate leaf bases that match the nuclear phylogenetic results. These inconsistent placements suggested potential hybridization events underlying the origins of *A. veitchianus* and *A. pycnophilus*. Notably, *Formania*, a shrub species nested within the shrub group clade in the plastid phylogeny (Clade P1d; Fig. 3), together with the three *Aster* species formed a well-supported clade as sister to the alpine *Aster* and *K. longipetiolata* (Clade N4; Fig. 3). Additionally, three shrub *Aster* species (*A. lavandulifolius*, *A. argyropholis* and *A. fulgidulus*) closely related to *A. albescens* and belonging to *A.* ser. *Albescentes* (*sensu*Ling, 1985) formed a basal branch of Clade N4 in the nuclear phylogeny (Fig. 4) with maximal support. The plastid and nuclear phylogenetic results showed a consistent pattern between the habitat and the phylogenetic placement of alpine *Aster* species, suggesting that these species should be excluded from Asterinae. Based on our phylogenetic results and the similar morphology and distribution, we here propose a new subtribe, Chlamyditinae, for the alpine *Aster*. Five genera were recently recognized to contain the alpine *Aster*: *Chlamydites* for three species with dense and white trichomes, *A. prainii*, *A. neolanuginosus* and *A. hololachnus* (Nesom, 2020*c*); *Griersonia* for *A. fuscescens* (not belonging to the Alpine *Aster* as defined in this study) and *A. senecioides*; *Geothamnus* for *A. batangensis* (Nesom, 2020*d*); *Iteroloba* for *A. bipinnatisectus* (Nesom, 2020*d*); and *Tibetiodes* for all the other alpine *Aster* species (Nesom, 2020*e*). Our results showed that *Chlamydites*, *Griersonia* and *Tibetiodes* were not monophyletic. We therefore proposed to treat the alpine *Aster* as one genus *Chlamydites*. Two genera, *Geothamnus* and *Tibetiodes*, should be reduced as synonyms, and *A. senecioides* should be transferred to *Chlamydites*.

While the plastid phylogeny failed to resolve the placement of *Aster fuscescens* with certainty (Fig. 3), nuclear phylogenetic results strongly supported its affinity with alpine *Aster* (Fig. 4). This species (including var. *oblongifolius*) has capitulescences composed of many densely arranged capitula – a morphological character shared with its nuclear phylogenetically determined sister species *A. senecioides*, showing congruence between phylogeny and morphology. Field observations of natural hybrids between *A. fuscescens* and *A. bietii*, i.e. *A. bietii* hybrid (Fig. 2), were observed in their sympatric distribution zone, further substantiating its evolutionary connection with the alpine *Aster* species.

*Aster bipinnatisectus* (=*Iteroloba bipinnatisecta*) was traditionally treated as a monotypic section of *Aster* and was recently separated from *Aster* (Nesom, 2020*d*) based mainly on its woody taproot and bipinnatisect leaves. Although not re-collected since its original description and absent from molecular analyses (including ours), its morphology of pappus and phyllaries suggests a potential affinity with the other alpine *Aster*. We provisionally maintain this taxon as *incertae sedis* due to insufficient phylogenetic and diagnostic morphological evidence. *Aster laka* (Nesom, 2020*f*), a western Himalayan alpine endemic recently treated as a close relative of *A. peduncularies* (the core *Aster* clade; Fig. 3), exhibits three diagnostic characters concordant with *Chlamydites*: (1) obligate alpine habitat, (2) herbaceous phyllaries and (3) biseriate pappus. While not sampled in our study, the morphological evidence supports its transfer from *Aster* to *Chlamydites*, pending molecular verification.

In the nuclear SNP phylogeny (Fig. 4), *Kalimeris longipetiolata*, *Formania mekongensis*, *Aster veitchianus*, *A. pycnophilus*, *A. lavandulifolius*, *A. argyropholis* and *A. fulgidulus* were resolved as closely related to alpine *Aster*, whereas they were nested within the core *Aster* clade in the plastid phylogeny (Fig. 3). Although *A. auriculatus* formed a close relationship with the alpine *Aster* in both nuclear and plastid analyses (Figs 3 and 4), it grouped with *A. veitchianus* and *A. pycnophilus* to form a well-supported clade in the nuclear SNP tree (Fig. 4). Given the conflicting phylogenetic positions of these species across the two datasets, they are provisionally treated here as Astereae species with unassigned subtribal placement.

### Phylogenetic position of the *Galatella* group and *Aster glehnii*

The *Galatella* group, containing three closely related genera, i.e. *Galatella*, *Crinitina* (=*Linosyris*) and *Tripolium*, was previously proposed as a natural group (Nesom, 1994*a*, *b*) characterized by its broad and multi-vein phyllaries generally with round tips (Nesom, 1994*a*, *b*). In this study, the *Galatella* group was monophyletic and with maximal support in both plastid and nuclear phylogenies (clades P1c and N5; Figs 3 and 4, respectively), corroborating earlier morphological and molecular studies (Brouillet *et al*., 2009; Jafari *et al*., 2015; Korolyuk *et al*., 2015). Our analyses strongly supported that *Tripolium* was the basal-most lineage of the group. Notably, *Aster glehnii*, an East Asian species recently segregated as *Kitamuria* (Nesom, 2020*g*), was placed as sister to the *Galatella* group with maximal support in both plastid and nuclear analyses (Figs 3 and 4), supporting the distant relationship between *A. glehnii* and typical *Aster* (clades P1a and N1a; Figs 3 and 4), and showing its close relationship with the *Galatella* group. In the plastid analysis, the *Galatella* group and *A. glehnii* were shown to have a relatively close relationship with other Asterinae members within Clade P1 (Fig. 3), whereas the nuclear phylogenetic results showed that these species were distant from the typical Asterinae species (Clade N1; Fig. 4). Our nuclear phylogenetic analysis supported that the *Galatella* group plus *A. glehnii* was the sister group of *Bellis*, supporting previous studies (Brouillet *et al*., 2009; Nesom, 2020*a*). However, because *Bellis* was not included in the plastid phylogenetic analysis and the placements of the *Galatella* group and *A. glehnii* were inconsistent in the plastid and nuclear analyses, whether the *Galatella* group and *A. glehnii* belong to Bellidinae was not fully tested here. Taxonomic treatments of these groups can be made on the basis of further phylogenetic analysis with expanded taxon sampling.

### Placement of the African *Aster* and the recognition of Afroasterinae

The African *Aster* group, containing 18 species, was previously treated as part of the genus *Aster* (Lippert, 1973; Nesom, 1994*b*; Nesom and Robinson, 2007). Brouillet *et al*. (2009) revealed that the African *Aster* species were not closely related to Eurasian *Aster* but were placed as a more basal lineage of tribe Astereae in a phylogenetic analysis using the ITS marker. Based on phylogenetic and morphological evidence, Manning and Goldblatt (2012) separated the African asters as a genus, i.e. *Afroaster*, and Nesom (2020*a*) raised this genus to a subtribe, i.e. Afroasterinae. Our results showed that the African asters were not members of Asterinae (Figs 3 and 4) and should be separated from *Aster*, which is consistent with the findings of Brouillet *et al*. (2009) and the subsequent separation of the genus (Manning and Goldblatt, 2012).

### Convergent evolution of pappus morphology in Asterinae and its implications in classification

The pappus, a key diagnostic character in Asteraceae, serves dual functions in achene protection and dispersal while exhibiting remarkable morphological plasticity. Within Asterinae, pappus type (Fig. 1) has been widely used in classification at the genus and species levels. Using our newly generated plastid phylogeny as the framework, we reconstructed evolutionary trajectories of pappus morphology across the subtribe (Fig. 6). Our results showed that the long pappus was a possible ancestral condition in the traditionally defined Asterinae. The other types were derived from the long pappus multiple times (Fig. 6), suggesting convergent evolution of the derived pappus types. Additionally, we detected at least four reversal transitions from the derived pappus types to the long pappus in the core Asterinae clade and the alpine clade. This recurrent homoplasy in pappus morphology (convergent gains/losses) fundamentally challenges traditional classification systems predicated on pappus characters. Our findings necessitate a paradigm shift toward integrative taxonomy combining molecular phylogenies with other morphological characters for robust systematic revisions in Asterinae. In addition, the alternative coding strategy with four independent characters provided the same evolutionary pattern with that of the single character coding strategy, suggesting the coding strategy did not affect the evolutionary analyses of the complex pappus morphology.

Traditional classifications of Asterinae relied heavily on pappus morphology for generic delimitation: *Kalimeris* was distinguished from *Aster* mainly by its short pappus, *Miyamayomena* by epappose achenes and *Heteropappus* by dimorphic pappus. However, our plastid phylogeny revealed that none of these three genera was monophyletic, and our ancestral state reconstruction based on pappus further showed that their particular pappus types convergently evolved from the long pappus shared by the majority of Asterinae species.

The *Heteropappus* complex exemplifies this evolutionary complexity. Our analyses placed all *Heteropappus* species in a clade with *Kalimeris shimadai* (short pappus) and *Aster handelii* (long pappus), showing one transition from the long pappus to the dimorphic pappus followed by two reversions to long pappus and one reduction to short pappus (Fig. 6). This pattern supports the inclusion of *Heteropappus semiprostratus* and *H. altaicus* within *Heteropappus* based on the similar morphology even though they both have a long pappus. Furthermore, *Aster handelii*, with a long pappus, and *Kalimeris shimadai*, with a short one, have a close relationship with *Heteropappus* in our phylogeny (Fig. 6). In addition, our analyses showed that the short pappus convergently evolved 14 times in the core Asterinae clade, supporting the non-monophyly of *Kalimeris*. Finally, pappus loss was detected in four lineages of the core Asterinae clade (Fig. 6), supporting the non-monophyly of *Miyamayomena*.

Our study here showed that the utility of the pappus in the definition of some Asterinae genera was generally not supported. Nevertheless, character analyses confirmed the taxonomic utility of the pappus in some clades. For example, *Sheareria nana* and the two species of *Miyamayomena* with epappose achenes formed a strongly supported clade. In the alpine clade, the double pappus was gained once by the ancestor of the alpine clade or the alpine clade except the earliest divergent lineage. Previously, these species with a double pappus were placed in different genera, and even the *Aster* species in this clade were previously grouped together with other species with a long pappus, such as *A. alpinus*. Our study showed that the double pappus accurately reflected their close phylogenetic relationships. Moreover, the double pappus observed in sampled *Kemulariella* species supported the closely related relationships revealed in the plastid phylogenetic analysis.

The pappus, a key structure for achene protection and dispersal in Asteraceae (Sheldon and Burrows, 1973; Andersen, 1993; Funk *et al*., 2009), exhibits a convergent evolutionary pattern in Asterinae. Our study reveals repeated independent reduction in pappus length across the subtribe, including shortening in *Kalimeris* and some species of *Aster*, and complete loss in *Miyamayomena* and *Sheareria*. While pappus reduction is associated with diminished dispersal efficiency and reduced seed germination rates in Asteraceae (Hale *et al*., 2010; Pliszko and Kostrakiewicz-Gierałt, 2020), paradoxically, many short pappus or epappose species of Asterinae thrive across broad geographical ranges.

Building on evidence that the pappus facilitates achene hydration in *Vernonia* (Shaukat *et al*., 2004), we also observed that Asterinae species with reduced pappus generally grow in humid environments. This correlation suggests a potential adaptive mechanism: in moisture-rich habitats, ambient humidity may ensure sufficient achene hydration, rendering the water-absorption function of long pappus redundant. Furthermore, species with reduced pappus (e.g. *Miyamayomena*, *Sheareria* and some *Aster*) are often restricted to localized humid environments (e.g. stream banks). We hypothesize that pappus reduction may limit dispersal to drier habitats, thereby favouring retention in optimal microenvironments – a selective pressure driving convergent pappus reduction across Asterinae. Notably, the achenes of *Sheareria* lost the pappus but developed an air cavity that may help the buoyancy of achenes (Gao *et al*., 2009), suggesting a potential shift in dispersal strategy from anemochory (wind-mediated) to hydrochory (water-mediated), further underscoring ecological context as a driver of pappus evolution. In summary, these findings highlight hydration dynamics, dispersal optimization and habitat specificity as interconnected factors shaping pappus reduction in Asterinae.

Our results showed that the double pappus evolved convergently three times within Asterinae, occurring in *Kemulariella*, *Callistephus* and the alpine *Aster* – all predominantly distributed in high elevations of alpine regions (Fig. 5). The double pappus developed an external row of scaly-like appendages that markedly increase pappus density. Given the extreme alpine conditions, we hypothesize that the double pappus may confer adaptive advantages over single-pappus forms by enhancing protection against the two key stressors: higher UV radiation and lower temperatures. This hypothesis aligns with prior evidence showing that trichome-rich structures mitigate UV damage in alpine plants (Karabourniotis *et al*., 1995, 2020; Yan *et al*., 2012) and improve thermal retention in cold environments (Meinzer and Goldstein, 1985; Cabrera *et al*., 1998; Peng *et al*., 2015). Such dual functionality could explain the repeated evolution of this character in alpine Asterinae lineages, reflecting a convergent adaptation to harsh high-elevation habitats.

## CONCLUSION

By reconstructing phylogenies using two datasets for Asterinae – five highly variable plastid DNA markers and 30 503 nuclear SNPs – we resolved the backbone phylogenetic relationships of the subtribe and revised its taxonomic circumscription. Our results reject the monophyly of previous morphologically defined Asterinae: most of its genera and species nested within a clade robustly supported by both plastid and nuclear phylogenies. This clade excludes several species (putatively of hybrid origin) and four genera that were previously placed in other subtribes or treated as unplaced groups of Astereae. Crucially, none of the three circumscriptions of *Aster* is monophyletic: *Aster s.l.*, *Aster s.s.* and the strictest circumscription of *Aster*.

Based on integrated phylogenetic and morphological evidence, we proposed recognizing only one genus *Aster* in Asterinae. This circumscription encompassed most *Aster* species, *Arctogeron*, *Asterothamnus*, Asian ‘*Doellingeria*’, *Heteropappus*, *Heteroplexis*, *Kalimeris* (except *K. longipetiolata*), *Miyamayomena*, *Rhynchospermum*, *Sheareria* and *Turczaninovia*. Our nuclear phylogenetic analysis further recognized four strongly supported subclades in the newly defined *Aster*, which we recognized as four subgenera of *Aster*. Notably, the African *Aster* (now as *Afroaster*), *Kemulariella* and alpine Asterinae species [including most members of *Aster* sect. *Alpigeni* (*sensu*Chen *et al*., 2011)] were resolved as distinct from the newly circumscribed Asterinae. *Aster glehnii* and the *Galatella* group (i.e. *Crinatina*, *Galatella* and *Tripolium*) formed a strongly supported monophyletic group and were placed as a clade distantly related to Asterinae in the nuclear phylogeny. Taxa with incongruent plastid–nuclear placements (three shrubby species of *A.* ser. *Albescentes*, *A. veitchianus*, *A. pycnophilus*, *A. auriculatus*, *A. fuscescens*, *Formania*, *Callistephus chinesis*, *Kalimeris cordiforlium*, *Neobrachyactis* and *Psychrogeton*) were excluded from the newly delimited *Aster*. This exclusion is provisional, pending further investigation with more nuclear loci.

Ancestral state reconstruction indicated that long pappus was ancestral in Asterinae and each of its four clades. Derived pappus types (e.g. reduced, double or lost pappus) evolved convergently on multiple occasions, with parallel pappus loss being the most common pattern in core Asterinae. While several reversions to the long pappus were detected within Asterinae, no regain of pappus occurred in epappose groups. These findings highlight the limited taxonomic utility of pappus morphology due to rampant homoplasy. However, double pappus can serve as one of the diagnostic characters for the *Kemulariella* clade and the alpine clade – two lineages that adapted to high-elevation environments. Additionally, our results showed that sister species often exhibit different pappus types, particularly within the core Asterinae clade. This suggested that pappus structure could be a useful character to distinguish closely related species.

## TAXONOMIC TREATMENT

**ASTERINAE** (Cass.) Dumort, Fl. Belg. Prodr. 66 (1827). Type: *Aster amellus* L. (Table 1; Fig. 6A–L) [紫菀亚族].

Annual, biennial or perennial herbs or subshrubs; stem leafy, or without true stems; leaves 1-nerved or 3-nerved, serrate or entire; involucres campanulate to narrowly campanulate; capitula generally several to many in corymbiform capitulescences, rarely solitary; phyllaries in 2–5 series, rarely more than 5 series, base hardened and apex herbaceous, outer phyllaries generally appressed, not keeled; achenes generally pubescent, glandular or not, generally 2–4 nerved, occasionally up to 10-nerved; pappus generally homogenous in all achenes with 1-series of bristles, equal to the length of disc corolla, sometimes reduced in ray floret achenes or all achenes, occasionally epappose or with an outer row of short scales.

This subtribe has ca. 125 species in one genus.

***Aster*** L., Sp. Pl. 2: 872 (1753). [Type: *Aster amellus* L.] (Fig. 6A–D) [紫菀属].

***Aster*** subg. ***Aster*** [紫菀亚属]

=*Kalimeris* (Cass.) Cass., Dict. Sci. Nat. 24: 324 (1822) [Type: *Kalimeris indica* (L.) Sch.Bip.]; ≡*Asteromoea* Blume, Bijdr. 901 (1826).

=*Rhynchospermum* Reinw., Syll. Pl. Nov. 2: 7 (1828) [Type: *Rhynchospermum verticillatum* Reinw.]

=*Heteropappus* Less., Syn. Gen. Compos. 189 (1832) [Type: *Heteropappus hispidus* (Thunb.) Less.]

=*Turczaninovia* DC., Prodr. [A. P. de Candolle] 5: 257 (1836) [Type: *Turczaninovia fastigiata* (Fisch.) DC.]

=*Arctogeron* DC., Prodr. (DC.) 5: 260 (1836). **syn. nov.** [Type. *Erigeron gramineum* L.]

=*Diplactis* Raf., Fl. Tellur. 2: 45 (1837) [Type: *Aster alpinus* L.]

=*Hisutsua* DC., Prodr. [A. P. de Candolle] 6: 44 (1838) [Type: *Hisutsua cantoniensis* (Lour.) DC.]

=*Sheareria* S.Moore, J. Bot. 13: 227 (1875), **syn. nov.** [Type: *Sheareria nana* S.Moore]

=*Martinia* Vaniot, Bull. Acad. Int. Géogr. Bot. sér. 3, 12(158): 31 (1902) [nom. illeg.], not Crié (1889) [Type: *Martinia polymorpha* Vaniot].

=*Heteroplexis* C.C.Chang, Sunyatsenia 3: 266 (1937), **syn. nov.** [Type: *Heteroplexis vernonioides* C.C.Chang]

=*Rhinactinidia* Novopokr., Trudy Bot. Inst. Akad. Nauk S.S.S.R., Ser. 1, Fl. Sist. Vyssh. Rast. 7: 114 (1948), **syn. nov.** [Type: *Rhinactinidia limoniifolia* (Less.) Novopokr. ex Botsch.]; ≡*Rhinactina* Less., Linnaea 6: 119 (1831) [nom. illeg.], not Willd. (1807); ≡*Krylovia* Schischk., in Krylov, Fl. Sibir. Occ., ed. 2, 11: 2670 (1949) [nom. illeg.], not Chachlov (1939); ≡*Borkonstia* Ignatov, Byull. Moskovsk. Obshch. Isp. Prir., Otd. Biol. 88(5): 105 (1983).

=*Asterothamnus* Novopokr., Bot. Mater. Gerb. Bot. Inst. Komarova Akad. Nauk S.S.S.R. 13: 331 (1950), **syn. nov.** [Type: *Aster alyssoides* Turcz.]

=*Miyamayomena* Kitam., Acta Phytotax. Geobot. 33: 409 (1982) [Type: *Miyamayomena savatieri* (Makino) Kitam.]; ≡*Gymnaster* Kitam., Mem. Coll. Sci. Kyoto Imp. Univ., Ser. B, Biol. 13: 301 (1982) [nom. illeg.], not F. Schütt (1891).

=*Yonglingia* Nesom, Phytoneuron 2020-57: 1 (2020). **syn. nov.**

=*Sinobouffordia* Nesom, Phytoneuron 2020-57: 15 (2020). **syn. nov.**

Herbs annual, biennial, perennial rhizomatous or subshrubs. Stem generally branched and leafy, sometimes with pseudoscapes and without true stems. Leaves basal and cauline; blade narrowly lanceolate to ovate, petiolate, serrate or rarely entire. Capitula radiate, in corymbiform capitulescences, rarely single. Involucres campanulate; phyllaries unequal, 3–5-seriate or sometimes more than 5 series, linear to lanceolate. Ray florets purple or white, generally more than 20, 1-seriate. Disc florets yellow or yellow-green, limb campanulate, with 5 shallowly split lobes, lobe not more than one-third of corolla length. Achenes cylindric or slightly compressed, 2–4-ribbed. Pappus 1-series of bristles equal to the length of corolla, sometimes reduced in ray floret achenes or all achenes, occasionally epappose.

This subgenus contains ∼110 species distributed in Eurasia (mainly East Asia) with one species reaching North America.

***Aster*** subg. ***Dongyaster*** G.J.Zhang. & T.G.Gao, **subg. nov.** [Type: *Aster scaber* Thunb.]; ≡*Cardiagyris* Nesom, Phytoneuron 2020-61: 1 (2020); *Aster* sect. *Teretachaenium* Kitam., Mem. Coll. Sci. Kyoto Univ., ser. B, 8: 3357 (1937) [东风菜亚属].

=*Aster* ser. *Sohayakienses* Kitam., J. Jap. Bot. 12 722 (1936) [Type: *Aster sohayakiensis* Koidz.]

=*Kalimeris* sect. *Cordifolium* Kitam., Mem. Coll. Sci. Kyoto Univ., ser. B. 8: 312 (1937) [Type: *Biotia japonica* Miq.]

Herbs perennial, rhizomatous. Stem simple, erect. Leaves basal and cauline; blade narrowly lanceolate to ovate, petiolate, serrate. Capitula, radiate, in corymbiform capitulescences, rare single. Involucres campanulate; phyllaries unequal, 3-seriate, lanceolate. Ray florets white, rarely purplish, generally less than 15, 1-seriate. Disc florets yellow or yellow-green, limb campanulate, with 5 deeply split lobes, lobe more than half of corolla length. Achenes cylindrical or slightly compressed, 3–10-ribbed. Pappus 1-series of bristles equal to the length of corolla, occasionally reduced.

*Aster komonoensis* was previously treated as a close relative of species of this subgenus (Nesom, 1993, 2020g; Ito and Soejima, 1995). However, on the basis of the amount of ray florets (generally more than 20), the purple colour of ray corolla and shallow disc floret lobes, we excluded it from *Aster* subg. *Dongyaster* and placed it in *A.* subg. *Aster*.

This subgenus contains seven species distributed in East Asia.

***Aster*** subg. ***Muwan*** G.J.Zhang. & T.G.Gao, **subg. nov.** [Type: *Aster poliothamnus* Diels] [紫菀木亚属];

Herbs with woody rhizomes or subshrubs. Stems many from rhizomes without obvious main stem, generally branched and leafy, sometimes with scapes and without true stems. Leaves generally cauline if plants with stems or leaves basal and forming rosette on the top of rhizome branches; blade linear, oblong or lanceolate, petiolate, serrate or rarely entire, generally hairy. Capitula radiate, single or in corymbiform capitulescences. Involucres campanulate to cylindrical; phyllaries equal in 2 series or unequal in 2–5 series, linear to lanceolate. Ray florets white or rarely purple, generally more than 20, 1-seriate. Disc florets yellow or yellow-green, limb campanulate, with 5 shallowly split lobes, lobe not more than one-third of corolla length. Achenes slightly compressed, 2–4-ribbed, generally hairy. Pappus 1-series of bristles equal to the length of corolla.

This subgenus contains around ten species distributed in China, Mongolia, Kazakhstan, Kirgizstan and Asian Russia.

***Aster*** subg. ***Liangwan*** G.J.Zhang. & T.G.Gao, **subg. nov.** [Type: *Aster hersileoides* C. K. Schneider] [亮菀亚属].

Shrubs. Stems with many branches, base of young branches generally surrounded by persistent scales. Leaves generally cauline; blade lanceolate to ovate, sessile to short petiolate, serrate or entire. Capitula radiate, single or in corymbiform capitulescences at top of branches. Involucres campanulate; phyllaries unequal, 3–4-seriate, linear to lanceolate. Ray florets purple, generally more than 20, 1-seriate. Disc florets yellow, limb campanulate, with 5 shallowly split lobes. Achenes slightly compressed, 2–4-ribbed, generally hairy. Pappus 1-series of bristles equal to the length of corolla.

This subgenus contains two species endemic to Sichuan and Guizhou provinces, and to Chongqing municipality of China.

### Generic names excluded from Asterinae

*Callistephus* Cass., Dict. Sci. Nat., ed. 2. 37: 491 (1825). [Type: *Aster chinensis* L.]; ≡*Callistemma* Cass., Bull. Sci. Soc. Philom. Paris 1817: 32 (1817), [nom. rej.]

*Crinitaria* Cass., Dict. Sci. Nat., ed. 2. 37: 463 (1825) [Type: *Crinitaria biflora* (L.) Cass.]

*Galatella* Cass., Dict. Sci. Nat., ed. 2. 37: 463 (1825). [Type: Galatella punctata (Waldst. & Kit.) Nees].

*Doellingeria* Nees, Gen. Sp. Aster. 177 (1832). [Type: *Doellingeria umbellata* (Mill.) Nees]

*Tripolium* Nees, Gen. Sp. Aster. 177 (1832). [Type: *Tripolium pannonicum* (Jacq.) Dobrocz.].

*Heterochaeta* DC., Prodr. 5: 282. 1836 [nom. illeg.], non Besser (1827). [Type: *Heterochaeta asteroides* DC.]

*Homostylium* Nees, Linnaea 18: 513 (1845) [Type: *Homostylium cabulicum* Nees.]

*Psychrogeton* Boiss., Fl. Orient. 3: 156 (1875). [Type: *Psychrogeton cabulicum* Boiss.]

*Chlamydites* J.R.Drumm., Bull. Misc. Inform. Kew 1907(3): 90 (1907). [Type: *Chlamydites prainii* J.R.Drumm.]

*Formania* W.W. Smith et J.Small, Trans. et Proc. Bot. Soc. Edinburgh 28: 91 (1922). [Type: *Formania mekongensis* W.W. Smith et J.Small.]

*Wardaster* J.Small, Trans. & Proc. Bot. Soc. Edin. 29: 230 (1926). [Type: *Wardaster lanuginosus* J.Small]

*Kemulariella* Tamamsch., Fl. URSS 25: 116 (1959). [Type: *Kemulariella caucasica* (Willd.) Tamamsch.]

*Crinitina* Soják, Zprávy Krajsk. Vlastiv. Muz. v Olomouci 215: 1 (1982) [Type: *Crinitina linosyris* (L.) Soják]; ≡*Linosyris* Cass., Dict. Sci. Nat., ed. 2. 37: 460 (1825) [nom. illeg.], not Ludw. (1757).

*Neobrachyactis* Brouillet, Fl. China 20-21: 571 (2011). [Type: *Neobrachyactis roylei* (DC.) Brouillet]

*Kitamuria* Nesom, Phytoneuron 2020-55: 1 (2020). [Type: *Aster glehnii* F.Schmidt].

*Sinosidus* Nesom, Phytoneuron 2020-64: 12 (2020) [Type: *Amphirhapis albescens* DC.] ≡ *Aster* ser. *Albescentes* Y.Ling, Fl. Reipubl. Popularis Sin. 74: 357, 179 (1985).

***Kemulariella*** group (Fig. 6M, N) [粉菀群].

Perennial herbs or subshrubs with leafy stems from rhizomes; leaves 1-nerved or 3-nerved, sessile; capitula solitary or rarely in loosely corymbiform capitulescences; phyllaries in 2–4 series, gradate to subequal, linear to lanceolate, herbaceous, slightly keeled; achenes 3-nerved, slightly compressed; pappus bristles in 2 unequal series, the outer series short and scale-like, the inner of bristles.

Our nuclear phylogenetic analysis showed that *Kemulariella* is not monophyletic. The clade circumscribed herein corresponds only to the type species of the genus *Kemulariella* Tamamsch, *K. caucasica*, with a distribution across the Caucasus and Turkey. Although the combined phylogenetic and morphological evidence strongly supported recognition of this clade at subtribal rank (as subtribe Kemulariellinae) (Figs 2 and 7), only two out of six species of the genus *Kemulariella* were sampled in this study. We adopted a conservative approach to avoid potential nomenclatural instability pending broader taxon sampling in future phylogenetic analyses. The placements of five other species, *K. abchasica*, *K. colchica*, *K. rosea*, *K. tuganiana* and *K. tahirelcii*, remain to be further studied. These taxa are currently treated as unplaced members of the Astereae tribe.

**Chlamyditinae** G.J. Zhang & T.G.Gao, **subtr. nov.** Type: *Chlamydites* J.R. Drummond [藏菀亚族]

Perennial herbs generally without true stem; single pseudoscape from the centre or side of basal leaf rosettes; leaves pubescent to villous, entire or rarely toothed; capitula solitary or rarely several in corymbiform capitulescences; involucres campanulate to salver-shape involucre; phyllaries in 2–3 series, generally equal in length, linear to lanceolate, fully herbaceous, not keeled, villous and generally glandular, outer phyllaries generally loose; achenes generally villous and glandular, 2–4-nerved; pappus bristles in 2 series, the outer of short and broad scales, the inner of bristles generally equal to the length of corolla.

We recognized one genus in this subtribe., *Chlamydites* J.R.Drummond, which contains all 29 alpine *Aster* species. *Aster fuscescense* [as the genus *Griersonia* of Nesom (2020*d*)] was shown to be a member of *Chlamydites* on the basis of the nuclear phylogeny. The recently separated *Tibetiodes* was treated as a synonym of *Chlamydites*. This subtribe contains ca. 30 species distributed in alpine areas of the Himalayas and Hengduan Mountains.

***Chlamydites*** J.R.Drummond, Bull. Misc. Inform. Kew 1907: 91 (1907). [Type: *Chlamydites prainii* J.R.Drummond] (Fig. 6Q–S) [藏菀属].

=*Wardaster* J.Small, Trans. Proc. Bot. Soc. Edinburgh 29: 230 (1926). [Type: *Wardaster lanuginosus* J.Small]

=*Tibetiodes* Nesom, Phytoneuron 2020-63: 1 (2020), **syn. nov.** [Type: *Tibetiodes diplostephioides* (DC.) Nesom]

=*Geothamnus* Nesom, Phytoneuron 2020-64: 1 (2020), **syn. nov.** [Type: *Geothamnus batangensis* (Bureau & Franch.) Nesom]

*Chlamydites* has ∼30 species mainly distributed in alpine areas of the Himalayas and Hengduan Mountains. Besides the type species *C. prainii*, 21 species previously placed in *Aster* were included in our phylogeny and 19 of them were transferred to this genus based on the phylogenetic and morphological evidence.

### Key to subtribes and groups recognized in the traditionally defined Asterinae and the four subgenera of *Aster* recognized in this study

1. Pappus of 2 series, with the outer series of short, broad scales and the inner series of bristles   2

1. Pappus of 1 series, with bristles only, sometimes reduced or absent 3 (Asterinae)

2. Plants without a true stem, pseudoscape arising from the centre or side of the basal leaf rosette, with 1 to few cauline leaves; phyllaries fully herbaceous, outer phyllaries generally loose Chlamyditinae

2. Plants with true stems, with many cauline leaves; the lower part of phyllaries hardened and the upper part herbaceous, outer phyllaries not loose *Kemulariella* group

3. Disc floret corolla deeply split, lobes more than half the length of the corolla; ray florets generally fewer than 15; achenes 3–10-ribbed *Aster* subg. *Dongyaster*

3. Disc floret corolla shallowly split, lobes not more than one-third the length of the corolla; ray florets generally more than 20; achenes 2–4-ribbed 4

4. Plants with woody rhizomes, subshrubs with obvious rays, or shrubs 5

4. Plants herbaceous or subshrubs without obvious rays *Aster* subg. *Aster*

5. Shrubs; base of young branches generally surrounded by persistent scales

*Aster* subg. *Liangwan*

5. Plants with woody rhizomes or subshrubs with obvious rays; base of young branches without persistent scales *Aster* subg. *Muwan*

### Taxa with uncertain subtribal position

***Neobrachyactis*** Brouillet, Fl. China 20-21: 571 (2011).

***Psychrogeton*** Boiss., Fl. Orient. [Boissier] 3: 156 (1875).

***Formania*** W.W. Smith et J.Small, Trans. et Proc. Bot. Soc. Edinburgh 28: 91 (1922).

***Callistephus*** Cass., Dict. Sci. Nat., ed. 2. 37: 491 (1825).

**‘*Aster*’ *auriculatus*** Franch., J. Bot. (Morot) 10: 376 (1896).

‘***Aster***’ (***Griersonia***) ***fuscescens*** Bureau & Franch., J. Bot. (Morot). 5: 49 (1891).

***‘Aster’* (*Iteroloba*) *bipinnatisectus*** Ludlow ex Grierson, Notes Roy. Bot. Gard. Edinburgh. 26: 144 (1964).

**‘*Aster*’ *pycnophyllus*** Franch. ex W.W.Smith, Notes Roy. Bot. Gard. Edinburgh 8: 332 (1915).

**‘*Aster*’ *veitchianus*** Hutch. et J.R.Drumm. ex G.J.Zhang & T.G.Gao, Phytotaxa 152: 53 (2013).

**‘*Aster*’ *albescens*** (DC.) Koehne, Deut. Dendrol. 562 (1893).

**‘*Aster*’ *argyropholis*** Hand.-Mazz., Acta Horti Gothob. 12: 208 (1938).

**‘*Aster*’ *fulgidulus*** Grierson, Notes Roy. Bot. Gard. Edinburgh 26: 110 (1964).

**‘*Aster*’ *hypoleucus*** Hand.-Mazz., J. Bot. 76: 285 (1938).

**‘*Aster*’ *lavandulifolius*** Hand.-Mazz., Notizbl. Bot. Gart. Berlin-Dahlem 13: 609 (1937).

‘***Aster***’ (***Cordiofontis***, ***Kalimeris***) ***longipetiolatus*** C.C.Chang, Sunyatsenia. 6: 22 (1941).

In our plastid and nuclear phylogenetic analyses, these genera and species exhibited incongruent placements. As their formal taxonomic treatment requiresfurther investigation, we tentatively assign them as subtribally unplaced taxa herein.

### Taxonomic treatments of species included in the present phylogeny

***Aster chenii*** H.H.Hu & T.G.Gao, **nom. nov.** [小花异裂菊]. Replaced name: *Heteroplexis microcephala* Y.L.Chen, Guihaia 5(4): 339 (1985), not *Aster microcephalus* (Miq.) Franch. (1875); the specific epithet honours Professor Yilin Chen, a distinguished Chinese taxonomist specializing in the classification of Astereae and several other angiosperm groups.

***Aster polii*** (Fanch.) G.J.Zhang & T.G.Gao, **comb. nov.** [虾须草]. Basionym: *Sheareria polii* Franch., J. Bot. 16: 257 (1878).

***Aster impressinervia*** (J.Y.Liang) H.H.Hu & T.G.Gao, **comb. nov.** [凹脉异裂菊]. Basionym: *Heteroplexis impressinervia* J.Y.Liang, Guihaia 14(2): 126 (1994).

***Aster liuzhouensis*** H.H.Hu & T.G.Gao, **nom. nov.** [柳州异裂菊]. Replaced name: *Heteroplexis incana* J.Y.Liang, Guihaia 14(2): 126 (1994), not *Aster incanus* (Lindl.) Jacques & Hérincq, Man. Gén. Pl. 2: 243 (1847); the specific epithet is derived from the type locality, Liuzhou City, Guangxi Zhuang Autonomous Region, China.

***Aster vernonioides*** (C.C.Chang) H.H.Hu & T.G.Gao, **comb. nov.** [异裂菊]. Basionym: *Heteroplexis vernonioides* C.C.Chang, Sunyatsenia 3: 267 (1937).

***Aster centraliasiaticus*** (Novopokr.) G.J.Zhang & T.G.Gao **comb. nov.** [中亚紫菀木]. Basionym: *Asterothamnus centraliasiaticus* Novopokr., Bot. Mater. Gerb. Bot. Inst. Komarova Akad. Nauk S. S. S. R. 13: 338 (1950).

***Aster gramineus*** (L.) G.J.Zhang & T.G.Gao **comb. nov.** [莎菀]. Basionym: *Erigeron gramineus* L., Sp. Pl. 2: 864 (1753).

***Chlamydites asteroides*** (DC.) G.J.Zhang & T.G.Gao **comb. nov.** [星舌藏菀]. Basionym: *Heterochaeta asteroides* DC., Prodr. 5: 282 (1836).

***Chlamydites batangensis*** (Bureau & Franch.) G.J.Zhang & T.G.Gao **comb. nov.** [巴塘藏菀]. Basionym: *Aster batangensis* Bureau & Franch., J. Bot. (Morot) 5: 50 (1891).

***Chlamydites bietii*** (Franch.) G.J.Zhang & T.G.Gao **comb. nov.** [线舌藏菀]. Basionym: *Aster bietii* Franch., J. Bot. (Morot) 10: 373 (1896).

***Chlamydites brachytrichus*** (Franch.) G.J.Zhang & T.G.Gao **comb. nov.** [短舌藏菀]. Basionym: *Aster brachytrichus* Franch., J. Bot. (Morot) 10: 372 (1896).

***Chlamydites bulleyanus*** (Jeffrey ex Diels) G.J.Zhang & T.G.Gao **comb. nov.** [扁毛藏菀]. Basionym: *Aster bulleyanus* Jeffrey ex Diels, Notes Roy. Bot. Gard. Edinb. 5: 184 (1912).

***Chlamydites diplostephioides*** (DC.) G.J.Zhang & T.G.Gao **comb. nov.** [重冠藏菀]. Basionym: *Heterochaeta diplostephioides* DC., Prodr. 5: 282 (1836).

***Chlamydites farreri*** (W.W. Smith & Jeffrey) G.J.Zhang & T.G.Gao **comb. nov.** [狭苞藏菀]. Basionym: *Aster farreri* W.W.Smith & Jeffrey, Notes Roy. Bot. Gard. Edinb. 9: 78 (1916).

***Chlamydites flaccidus*** (Bunge) G.J.Zhang & T.G.Gao **comb. nov.** [萎软藏菀]. Basionym: Aster flaccidus Bunge, Mém. Acad. Imp. Sci. St.-Pétersbourg Divers Savans 2: 599 (1835).

***Chlamydites himalaicus*** (C.B.Clarke) G.J.Zhang & T.G.Gao **comb. nov.** [须弥藏菀]. Basionym: *Aster himalaicus* C.B.Clarke, Comp. Ind., 42 (1876).

***Chlamydites hololachnus*** (Y.L.Chen, S.Yun Liang & K.Y.Pan) G.J.Zhang & T.G.Gao **comb. nov.** [全茸藏菀]. Basionym: *Aster hololachnus* Y.Ling ex Y.L.Chen, S.Yun Liang & K.Y.Pan, Acta Phytotax. Sin. 19(1): 88 (1981).

***Chlamydites jeffreyanus*** (Diels) G.J.Zhang & T.G.Gao **comb. nov.** [滇西北藏菀]. Basionym: *Aster jeffreyanus* Diels, Notes Roy. Bot. Gard. Edinb. 5: 185 (1912).

***Chlamydites likiangensis*** (Franch.) G.J.Zhang & T.G.Gao **comb. nov.** [丽江藏菀]. Basionym: *Aster likiangensis* Franch., J. Bot. (Morot) 10: 370 (1896).

***Chlamydites ruoqiangensis*** (Y.Wei & C.H.An) G.J.Zhang & T.G.Gao **comb. nov.** [若羌藏菀]. Basionym: *Aster ruoqiangensis* Y.Wei & C.H.An, Fl. Xinjiangensis 5: 475 (1999).

***Chlamydites salwinensis*** (Onno) G.J.Zhang & T.G.Gao **comb. nov.** [怒江藏菀]. Basionym: *Aster salwinensis* Onno, Biblioth. Bot. 26 (Heft 106): 74 (1932).

***Chlamydites senecioides*** (Franch.) G.J.Zhang & T.G.Gao **comb. nov.** [狗舌藏菀]. Basionym: *Aster senecioides* Franch., J. Bot. (Morot). 10: 381 (1896).

***Chlamydites setchuenensis*** (Franch.) G.J.Zhang & T.G.Gao **comb. nov.** [四川藏菀]. Basionym: *Aster setchuenensis* Franch., J. Bot. (Morot) 10: 377 (1896).

***Chlamydites stracheyi*** (Hook. f.) G.J.Zhang & T.G.Gao **comb. nov.** [匐生藏菀]. Basionym: *Aster stracheyi* Hook. f., Fl. Brit. India 3(8): 250 (1881).

***Chlamydites tientschwanensis*** (Hand.-Mazz.) G.J.Zhang & T.G.Gao **comb. nov.** [天全藏菀]. Basionym: *Aster tientschwanensis* Hand.-Mazz., Oesterr. Bot. Z. 89: 819 (1940).

***Chlamydites yunnanensis*** (Franch.) G.J.Zhang & T.G.Gao **comb. nov.** [云南藏菀]. Basionym: *Aster yunnanensis* Franch., J. Bot. (Morot) 10: 375 (1896).

## Supplementary Material

mcag225_Supplementary_Data

## Data Availability

The new sequences generated using Sanger sequencing have been deposited in GenBank with the accession numbers given in Supplementary Data Table S1. The raw sequences generated using RADseq have been deposited in the GSA platform (No. CRA040592) of National Genomic Data Center (NGDC), China National Center for Bioinformation (CNCB) with accession numbers shown in Supplementary Data Table S2.
