# Supplementary material for: Phylogeny of the subtribe Asterinae (Asteraceae: Astereae): new circumscription for Asterinae and Aster and implications for pappus evolution
Source: Ann Bot. 2026 Jul 25;138(5):1779–803. doi: 10.1093/aob/mcag225 (PMC13715008; doi:10.1093/aob/mcag225)
Supplement: Supplementary file 1 [file mcag225_supplementary_data.zip › SupFigs1-3.pdf]

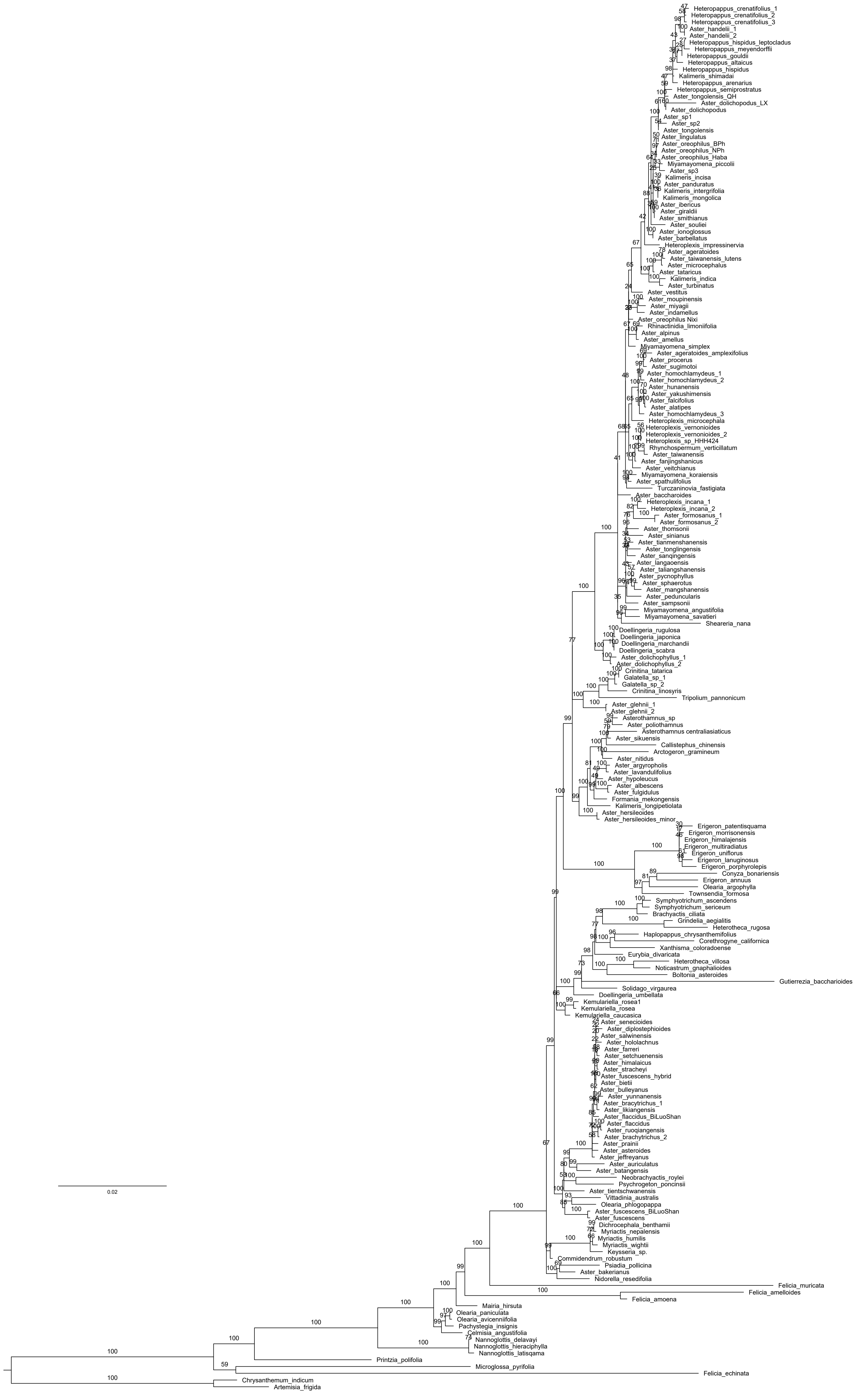

Supplemental Figure S1. Phylogenetic tree of Asterinae reconstructed using IQ-TREE with partitioned models.

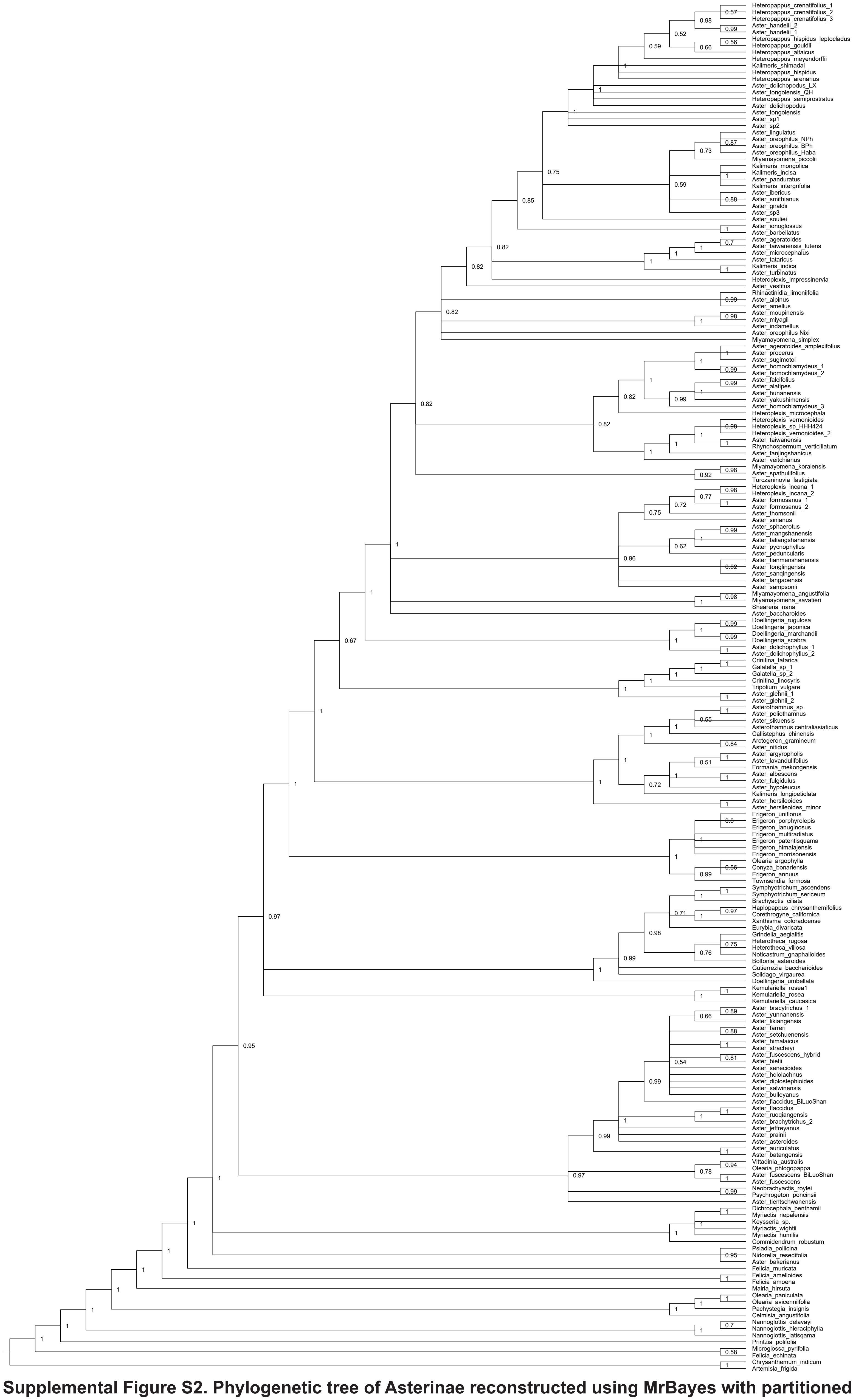

Supplemental Figure S2. Phylogenetic tree of Asterinae reconstructed using MrBayes with partitioned models.

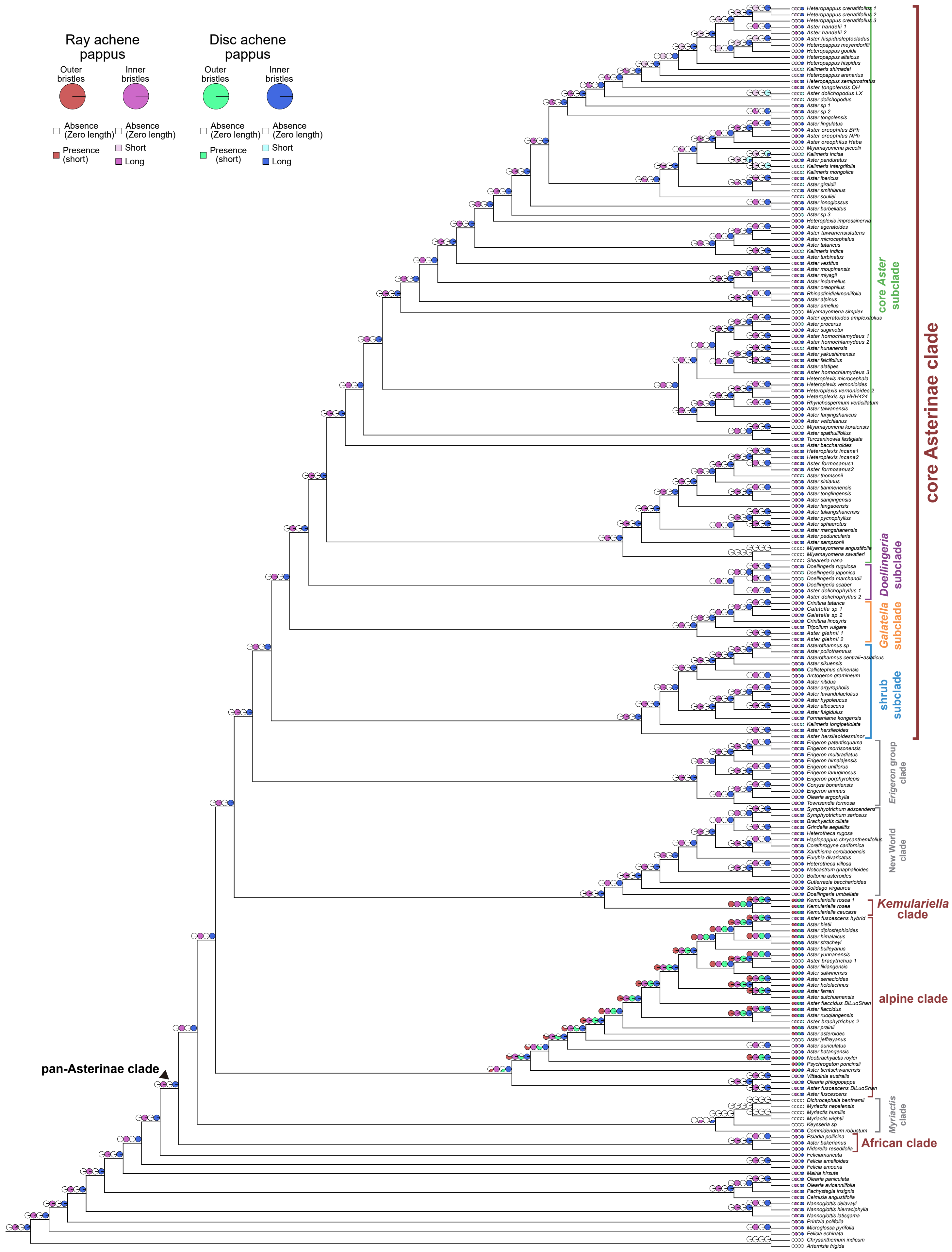

Supplemental Figure S3. Ancestral state reconstruction of pappus morphology using independent coding strategy. The pappus morphology was divided into four independent characters (from left to right in each node), i.e., (1) presentness of outer bristles in achenes of ray florets, (2) length of inner bristles in achenes of ray florets, (3) presentness of outer bristles in achenes of disc florets, (4) length of inner bristles in achenes of disc florets.
